# CIDNP study of photoinduced electron transfer in His-Glu-Tyr-Gly peptide and its conjugate His-Gln(BP)-Tyr-Gly

**DOI:** 10.1038/s41598-025-04831-6

**Published:** 2025-06-20

**Authors:** Natalya N. Fishman, Kevin Herr, Olga B. Morozova, Ivan V. Zhukov, Maksim P. Geniman, Martin Brodrecht, Till Wissel, Gerd Buntkowsky, Alexandra V. Yurkovskaya

**Affiliations:** 1 International Tomography Center, Institutskaya 3a, 630090 Novosibirsk, Russia; 2 Institute of Physical Chemistry, Technical University Darmstadt, Peter-Grünberg-Str. 8, 64287 Darmstadt, Germany; 3 Novosibirsk State University, 2 ul. Pirogova, 630090 Novosibirsk, Russia

**Keywords:** Biophysical chemistry, NMR spectroscopy, Solution-state NMR

## Abstract

Photoinduced intramolecular electron transfer (ET) is essential for understanding charge transport in biological and synthetic systems. This study examines ET in peptide His-Glu-Tyr-Gly (1) and the conjugate His-Gln(BP)-Tyr-Gly (2) with benzophenone (BP) as a photoactive electron acceptor and His or Tyr as donors. Time-resolved and field-dependent chemically induced dynamic nuclear polarization (CIDNP) techniques were employed to investigate ET mechanisms and kinetics. Peptide 1 with 3,3’,4,4’-tetracarboxy benzophenone as a photosensitizer initially forms two types of radical with radical center at either His or Tyr residue, the consequent intra- and intermolecular ET electron transfer from Tyr residue to the His radical takes place with rate constants k_e(intra)_=(1.5±0.5)×10^5^ s^− 1^ and k_e(inter)_=(1.3±0.4)×10^7^ M^− 1^s^− 1^ at pH 8.8. Conjugate 2 forms two types of biradicals under irradiation: with radical centers at Tyr and BP across the entire pH range, and with radical centers at His and BP at slightly basic pH. Field-dependent CIDNP revealed nonzero electronic exchange interaction (2J_ex_ = − 8.78 mT) at acidic pH, indicating proximity between BP and Tyr radicals. Low-field CIDNP spectra showed strong emissive polarization patterns, with pH-dependent exchange interaction and biradical geometry. Notably, no electron transfer from tyrosine to histidine radicals was observed in the conjugate 2, distinguishing its behavior from peptide 1.

**Supplementary Information:**

The online version contains supplementary material available at 10.1038/s41598-025-04831-6.

## Introduction

The transfer of electrons (ET) over long distances in living cells is a crucial process in a vast array of biochemical activities, including respiration and photosynthesis^1–4^. Despite the extensive study of numerous model peptide systems^5–10^, it is evident that the kinetics of the process are influenced by the molecular dynamics of the system and a number of molecular structure parameters, including donor/acceptor distance, amino acid sequence, terminal amino group charge, and reactant protonation state. Further investigation is needed to define a clear mechanism and fully understand the role of these and other factors. Photoinduced electron transfer is a special case of electron transfer. It is a light-driven process that forms a charge-separated state consisting of a donor radical cation, D^•+^, and an acceptor radical anion, A^•−^. In an intramolecular photoinduced ET process, D and A are part of the same molecule. In an intermolecular photoinduced ET process, they belong to different molecules. Photoinduced ET occurs in many chemical and biological scenarios, including photovoltaic devices, molecular photo-switches, and natural and artificial photosynthesis^11–16^. The electrochemical and excited state properties of the acceptor and donor moieties modulate photoinduced ET. However, it is important to consider that charge separation or recombination (back electron transfer) can be influenced by weak interactions of electron spins with each other, with a magnetic field and spin of surrounding nuclei, which is often neglected because thermodynamically they are of minor importance. This interaction significantly changes the quantum yield of the photoreaction. The cation of the donor radical and the anion of the acceptor radical interact electrostatically and magnetically. The strength of this interaction is determined by three factors: the distance between the radicals, their relative orientation, and, in the case of intramolecular ET, the nature of the linker connecting the radicals. The recombination rate of the singlet and triplet charge separation states differs significantly. The back electron transfer reaction from the singlet state is much faster than from the triplet state. The transition between these states is forbidden due to the difference in the total spin quantum number. However, spin interactions of electrons with each other, with an external magnetic field, and with spins of magnetic nuclei remove this spin prohibition and induce intersystem crossing transitions between singlet and triplet states. This has an important effect on the lifetime of the charge separation state. Studying magnetic and spin effects in the reversible electron transfer reaction allows to exploit the controlling effect of very weak interactions of electron spins with external magnetic fields and surrounding nuclei^17–21^.

While researchers in various fields of science have shown great interest in intramolecular photoinduced ET processes, these processes have been less extensively studied compared to intermolecular processes. This is particularly true for processes occurring in living organisms and in various model systems that simulate them. Biomimetic models designed for this purpose include the Tyr/Trp and His/Tyr pairs, that serve as an illustrative example of a redox-active site found in natural enzymes^6,7,22–27^. It is established that Tyr residues facilitate long-range electron transfer processes in a number of enzymes^11,28–32^. Tyr exhibits a lower oxidation potential (0.9 V)^33^ than His (1.17 V)^34^. However, in the presence of chemical agents with high oxidizing ability (e.g., photo-excited benzophenone derivatives), both His and Tyr residues can undergo oxidation. When the initial His^•^ radical is formed, the intramolecular electron transfer from Tyr to His^•^ occurs^35–37^. Whereas the reaction with the participation of Tyr in ET processes are mostly studied by time-resolved methods of laser pulsed photolysis or EPR spectroscopy^4,6,7,38^, it is difficult or almost impossible to study directly the reactions involving the histidine radicals using these methods: histidine radicals are poor chromophores which precludes their optical detection, and they are too short-lived at ambient conditions which does not allow them to be detected by EPR. In investigating the structure and properties of transient histidine radicals in the presence of other amino acid the chemically induced dynamic nuclear polarization (CIDNP) method plays a pivotal role^35,36,39,40^. By employing UV irradiation directly within the NMR spectrometer probe, the CIDNP technique enables the characterization of paramagnetic species generated during irradiation^36,41,42^. CIDNP is manifested by NMR signals with non-equilibrium nuclear spin populations − either enhanced absorption or emission − arising in nuclei with spin density of an unpaired electron in intermediate paramagnetic particles, which serve as precursors to polarized diamagnetic compounds^43,44^. Analysis of CIDNP signal patterns provides valuable information on the signs of hyperfine coupling constants (HFCCs), differences in g-factors of paramagnetic species, and other parameters essential for elucidating radical reaction mechanisms^42,45,46^. Previously, a time-resolved version of CIDNP was employed to investigate the kinetics of electron transfer from tyrosine to short-lived histidine radicals in peptides of varying structures^35,36^. Furthermore, the complete reaction scheme of histidine radical reduction by tyrosine for free amino acids and their N-acetylated derivatives was established, and electron transfer was identified as the reaction mechanism in the pH range from 6 to 12^40^. The influence of the protonation state of the reactants on the reaction rate constants was studied in detail, and it was found that the reduction of the histidine radical by tyrosine is always irreversible.

**Fig. 1:**
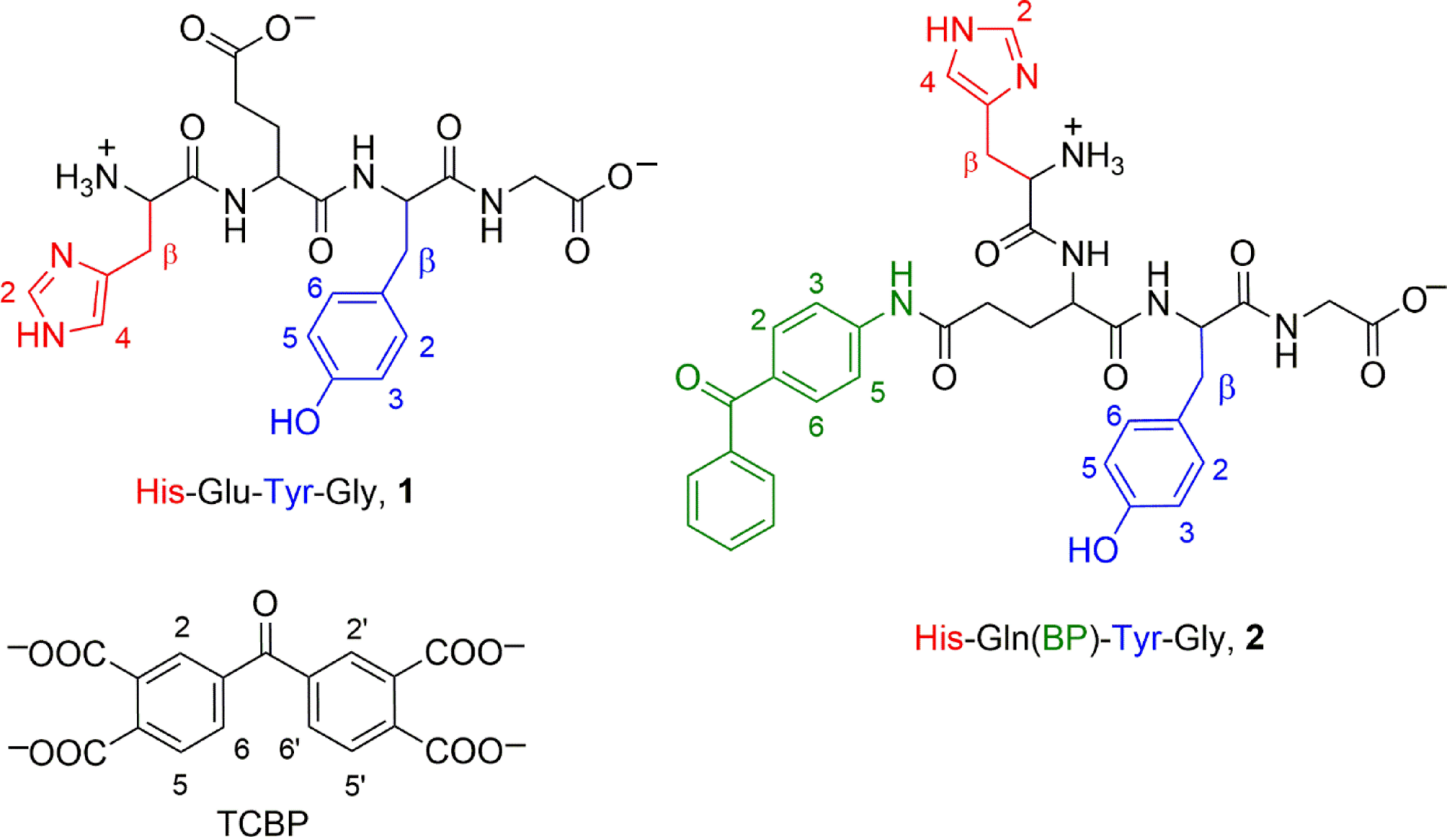
Structures of the His-Glu-Tyr-Gly peptide **1**, 3,3’,4,4’-tetracarboxy benzophenone TCBP, and His-Gln(BP)-Tyr-Gly conjugate **2**, in neutral aqueous solution.

The aim of this work was to reveal and characterize photoinduced intramolecular ET reactions in model objects, synthetic tetrapeptide **1** − His-Glu-Tyr-Gly, and newly developed tetrapeptide/photosensitizer conjugate **2** − His-Gln(BP)-Tyr-Gly (Fig. 1). In this conjugate we have introduced the photosensitizer 4-aminobenzophenone by conjugation with glutamate midway between the histidine (at the N-terminus) and tyrosine moieties. In such a system, intramolecular ET is initiated by UV light and occurs between the photoexcited triplet benzophenone and the amino acid residue with the low oxidation potential. The peptide **1** can only undergo intermolecular benzophenone-sensitized photoinduced oxidation. Both of these biomimetic chemical compounds can be used as model systems to provide detailed mechanistic investigation of the underlying elementary reaction steps that are of biological relevance.

Two variations of the CIDNP method were employed: time-resolved and field dependent. Time-resolved CIDNP has been used to study the photoinduced oxidation of peptide **1** by triplet-excited 3,3’,4,4’-tetracarboxy benzophenone (TCBP, Fig. 1). A combination of time-resolved and field-dependent CIDNP techniques was applied to the study of photo-induced reactions of the peptide/photosensitizer conjugate **2**. The details of these experimental methods are summarized in Materials and Methods Section. The CIDNP technique allows the sensitive detection not only of transient mono-radicals but also biradicals even if the biradicals cannot be observed by EPR methods due to their short lifetime. Furthermore, CIDNP is capable of detecting biradicals in liquids at ambient temperature. The reason for this is that CIDNP is formed on the nanosecond to microsecond time scale and persists during typical nuclear relaxation time in diamagnetic molecules, providing a “frozen signature“^41^ of the elusive radicals. Analyzing the CIDNP as a function of time and magnetic field allows to determine the reaction mechanism, the rate constants of the radical reactions and the magnetic resonance parameters of such radicals. At present, the study of the CIDNP field dependence of the products of biradical conversion is the most reliable indirect method for demonstrating the involvement of biradicals in chemical reactions. Previously, it was shown that CIDNP kinetics in biradicals can provide information about the competition between spin dynamics, the chemical reaction of decarboxylation, which transforms primary radicals into secondary radicals, and the molecular dynamics of a linker^47–54^.

In the present study we (1) analyzed CIDNP kinetic data obtained for the photoreaction of TCBP and peptide **1** with the aim to determine the rate constant of photoinduced intramolecular ET in peptide **1**; (2) measured CIDNP kinetics in the photoreaction of conjugate **2** in order to determine the pathways of the primary photochemical reaction of triplet benzophenone quenching and to identify the contribution of the potential intramolecular ET from a tyrosine residue to a histidine radical; (3) measured CIDNP field dependences in photoreaction of conjugate **2** to reveal the biradicals formation and characterize their magnetic properties.

## Results and discussion

### Selection of experimental conditions

It is known from previous studies that the processes of photoinduced oxidation of histidine and tyrosine^55^, as well as the electron transfer from tyrosine to the histidine radical^36,40^, are determined by the protonated state of the reactants. In order to obtain the p*K*_a_ values that determine the protonated states (SI, Figure S1), a chemical shift titration of the conjugate **2** was performed. It turned out that conjugate **2** with neutral imidazole and charged amino group is poorly soluble in water and precipitated during titration, therefore experiments were possible only at pH below p*K*_a_ of imidazole (typical value ~ 6 for N-terminal histidine) or above p*K*_a_ of amino group (typical value ~ 7.6). Thus, pH values of ~ 4 and ~ 8.7 (and higher) were chosen. Accordingly, to determine the rate constant of intramolecular electron transfer in oxidized peptide **1**, experiments were carried out at pH 8.8. The only acidity constant that could be determined was that for phenol of tyrosine residue: p*K*_a_=10.8 (see Materials and Methods Section for details).

### Electron transfer from Tyr residue to His radical in His-Glu-Tyr-Gly peptide

As in our previous studies, radicals were generated in the photoinduced reaction with the photosensitizer 3,3’,4,4’-tetracarboxy benzophenone, rather than 4-aminobenzophenone, which was employed in the synthesis of conjugate **2**. This was due to the low solubility of 4-aminobenzophenone in water. CIDNP effects in the photochemical reaction of TCBP with His and Tyr and manifestation of the reduction reaction in the CIDNP kinetics are described in detail in our previous publications^36,40,55^ and are only briefly summarized below. In the photoreaction with triplet-excited TCBP, two types of peptide radicals can be formed, with a radical center at either the His residue or the Tyr residue. As demonstrated in our previous studies, histidine and tyrosine exhibit comparable reactivity towards triplet-excited TCBP at neutral and slightly basic pH^40^. The reaction mechanism for both amino acids at neutral and slightly basic pH is proton-coupled electron transfer (PCET)^56^. At pH 8.8, the reactions leading to the triplet radical pairs (RP) formation are as follows (here the superscript T denotes the triplet state of the radical pair under overbar):

1 $$^{{\text{3}}}{\text{TCBP }}+{\text{ HisH}}-{\text{Glu}}-{\text{TyrOH}}-{\text{Gly}} \to {}_{{}}^{{\text{T}}}\overline {{{\text{TCBP}}{{\text{H}}^ \bullet }~~~{\text{~~Hi}}{{\text{s}}^ \bullet}-{\text{Glu}}-{\text{TyrOH}}-{\text{Gly}}~}}$$

2 $$^{{\text{3}}}{\text{TCBP }}+{\text{ HisH}}-{\text{Glu}}-{\text{TyrOH}}-{\text{Gly}} \to {}_{{}}^{{\text{T}}}\overline {{{\text{TCBP}}{{\text{H}}^ \bullet }~~~~~{\text{HisH}}-{\text{Glu}}-{\text{Tyr}}{{\text{O}}^\bullet }-{\text{Gly}}~}} .$$

**Fig. 2:**
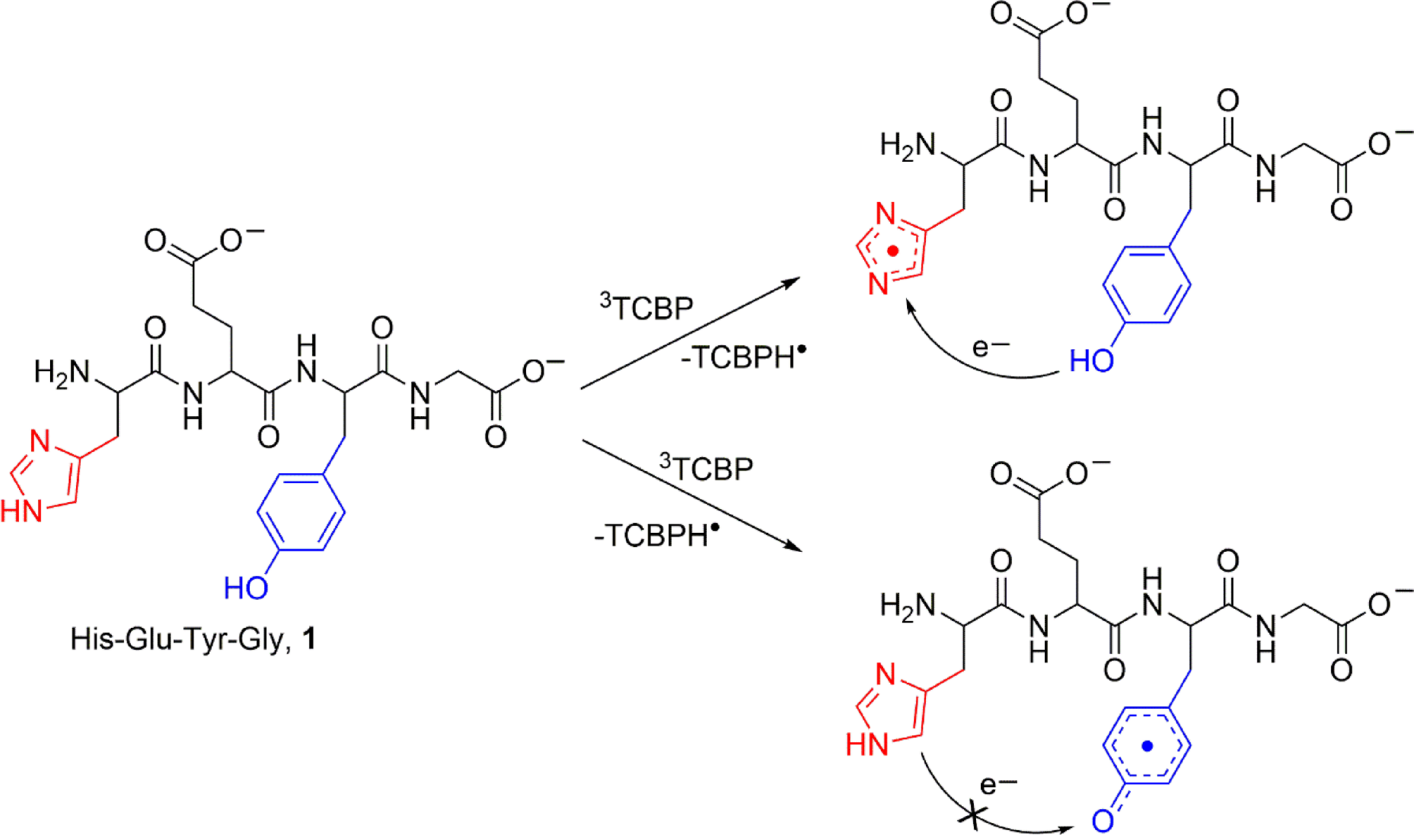
Two types of peptide **1** radicals formed in the photoinduced reactions of 3,3′,4,4′-tetracarboxy benzophenone and His-Glu-Tyr-Gly (HisH-Glu-TyrOH-Gly) in slightly basic solution. Intramolecular (as well as intermolecular) electron transfer from Tyr to His^•^ occurs, whereas TyrO^•^ radical is not reduced by histidine.

Here H in HisH refers to imidazolyl proton, and OH in TyrOH refers to phenyl moiety. The two types of radical pairs are formed (shown in Fig. 2), then eventually recombine back to the diamagnetic initial compounds. This process leads to characteristic polarized NMR signal patterns of His, Tyr and TCBP. By recording the polarized NMR spectra at a certain delay after the laser flash that initiates the reaction, the kinetics of CIDNP can be obtained at the time-scale from one microsecond to about 100 microseconds (this is referred to as the ‘kinetic window’ of the method), applying the technique known as time-resolved CIDNP^41,57–61^. It is an elegant method to study intra- and intermolecular ET reactions in peptides^36,62^. This technique allows the identification of transfer pathways and precise determination of electron transfer rates, which is beneficial for understanding the mechanisms of peptide radical formation.

**Fig. 3:**
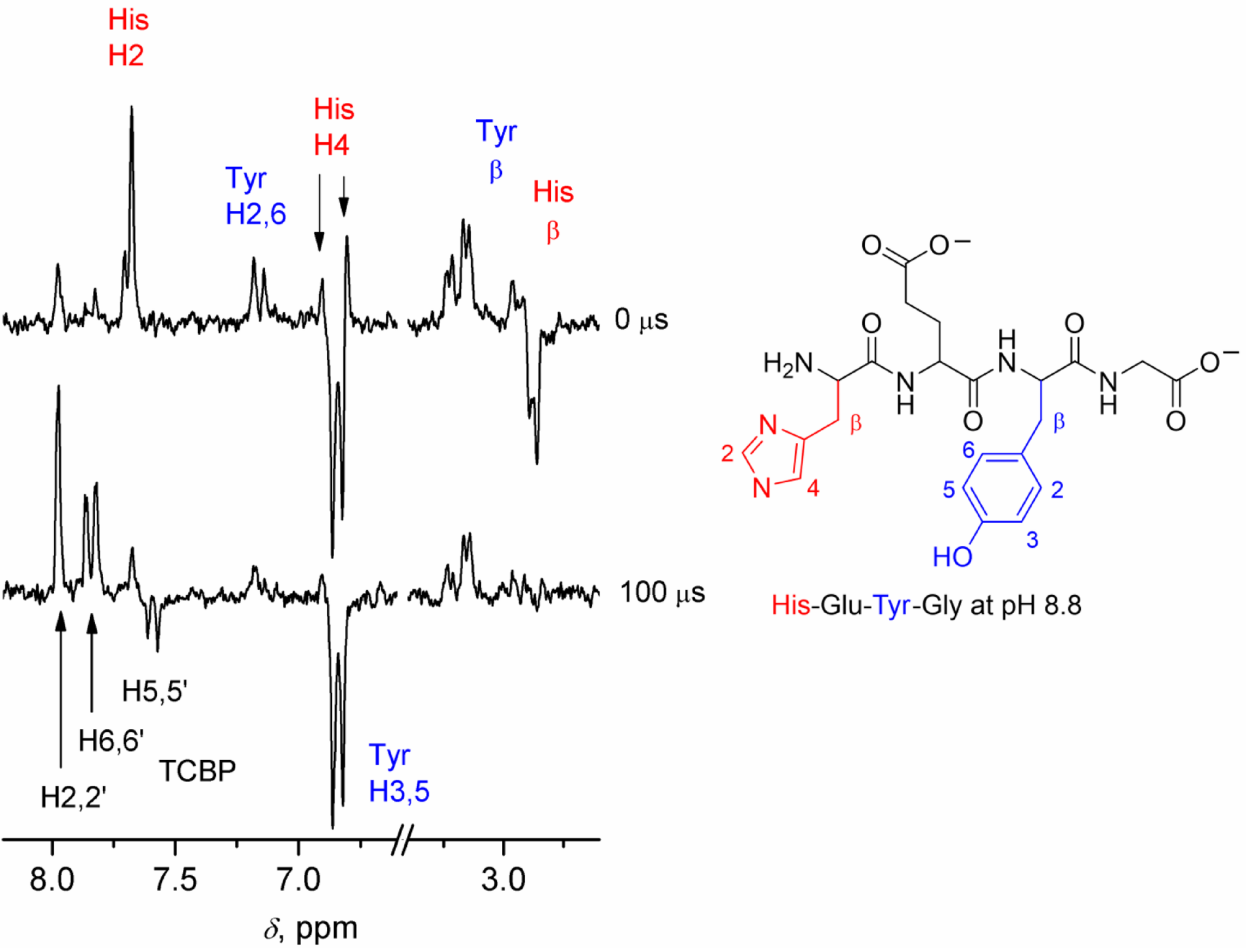
^1^H CIDNP spectra obtained for the photoreaction of 2 mM TCBP with 40 mM His-Glu-Tyr-Gly at pH = 8.8 at 0 µs (top) and 100 µs (bottom) after the laser pulse. The aromatic protons of histidine are represented by signals of two stereoisomers.

Figure 3 shows the CIDNP spectra obtained for the photoreaction of TCBP with HisH-Glu-TyrOH-Gly at pH 8.8. The upper spectrum, obtained without delay following the laser flash, contains CIDNP signals from His and Tyr residues, as well as from TCBP as reversible reaction products formed at the geminate reaction stage. The CIDNP signals are in accordance with Kaptein’s rule for the net CIDNP effect^63^. According to this rule, the CIDNP sign for the product of geminate recombination is determined by the sign of the HFCC of the nucleus under consideration in the radical, by the difference between the g-factor of this radical and of the partner radical, and by the precursor multiplicity (triplet in our case). TCBP radical^64^ has a g-factor that is greater than that of the histidine radical^65^, and less than that of the tyrosine radical^66^. Thus, enhanced absorption occurs for protons with positive HFCCs in Tyr radical, and negative HFCCs in His radical: H2, H4 protons of His, H2,6, and β protons of Tyr^67^. Emission occurs for protons with positive HFCCs in His radical, and negative HFCCs in Tyr radical: β protons of His, H3,5 of Tyr^67^. The polarization of TCBP formed in radical pairs with a His radical is of an opposite sign to that formed in radical pairs with a Tyr radical. The resulting CIDNP of TCBP protons is determined by (i) the proportion of peptide radicals of each type (with radical center at histidine or tyrosine residue) formed in the quenching reaction, and (ii) the ratio of the CIDNP enhancement factors for TCBP protons in the two types of radical pairs. The initial positive polarization of H2,2’ and H6,6’ and negative polarization of H5,5’ of TCBP reflects predominance of CIDNP formed in a radical pair with Tyr radical, the same as in the photoreaction of a dye with peptides of different structures containing His and Tyr residues^56^.

Our preceding study^36^ provided a solid background for determining intramolecular ET rate constant in the oxidized peptide **1**. In that study, the pH-dependences of the observed ET rate constant were analyzed for peptides of different structures at concentrations 20 and 40 mM. Intramolecular ET equally contributes at both concentrations, while intermolecular ET contribution is concentration-dependent. At the pH = 8.8 chosen for this study, Tyr is mainly present in solution with neutral phenol (TyrOH, p*K*_a_=10.8), the proportion of tyrosine in the form of tyrosinate anion is ~ 1%. However, the rate constant of intermolecular ET from tyrosinate was found to be about two orders of magnitude higher, than that from neutral tyrosine, and contributes to the total intermolecular ET at pH 8.8. No intramolecular ET from tyrosinate anion was revealed. In the case of neutral tyrosine, both intramolecular and intermolecular electron transfer reactions were found to occur.

The histidine radical is present in neutral and basic aqueous solutions in its neutral form with an unpaired electron localized on the imidazole ring^65,68^. The reduction of the His radical leads to the formation of the His anion, which protonates rapidly in the reaction with water (p*K*_a_ of HisH > 14)^68^. Cation radical of Tyr formed after ET quickly deprotonates^69^. Thus, the net intramolecular ET reaction involves neutral phenol and imidazole (as product), and is as follows:

3 $${\text{Hi}}{{\text{s}}^ \bullet }-{\text{Glu}}-{\text{TyrOH}}-{\text{Gly}}~\xrightarrow{{{k_{{\text{e~}}\left( {{\text{intra}}} \right)}}}}{\text{HisH}}-{\text{Glu}}-{\text{Tyr}}{{\text{O}}^ \bullet }-{\text{Gly}}$$

The corresponding net intermolecular reactions at pH 8.8 are:

4 $$\begin{array}{*{20}{c}} {{\text{Hi}}{{\text{s}}^ \bullet }-{\text{Glu}}-{\text{TyrOH}}-{\text{Gly}}} \\ {+\,{\text{HisH}}-{\text{Glu}}-{\text{TyrOH}}-{\text{Gly}}} \end{array}\xrightarrow{{{k_{{\text{e1~}}\left( {{\text{intra}}} \right)}}}}\begin{array}{*{20}{c}} {{\text{HisH}}-{\text{Glu}}-{\text{TyrOH}}-{\text{Gly }}} \\ {+{\text{ HisH}}-{\text{Glu}}-{\text{Tyr}}{{\text{O}}^ \bullet }-{\text{Gly}}} \end{array}$$

5 $$\begin{array}{*{20}{c}} {{\text{Hi}}{{\text{s}}^ \bullet }-{\text{Glu}}-{\text{Tyr}}{{\text{O}}^ - }-{\text{Gly}}+\,{\text{HisH}}\,} \\ {-{\text{Glu}}-{\text{Tyr}}{{\text{O}}^ - }-{\text{Gly}}\,+\,{{\text{H}}_{\text{2}}}{\text{O}}} \end{array}\xrightarrow{{{k_{{\text{e2~}}\left( {{\text{intra}}} \right)}}}}\begin{array}{*{20}{c}} {{\text{HisH}}-{\text{Glu}}-{\text{Tyr}}{{\text{O}}^ - }-{\text{Gly}}\,+\,{\text{HisH}}} \\ {-{\text{Glu}}-{\text{Tyr}}{{\text{O}}^ \bullet }-{\text{Gly}}\,+\,{\text{O}}{{\text{H}}^ - }} \end{array}$$

**Fig. 4:**
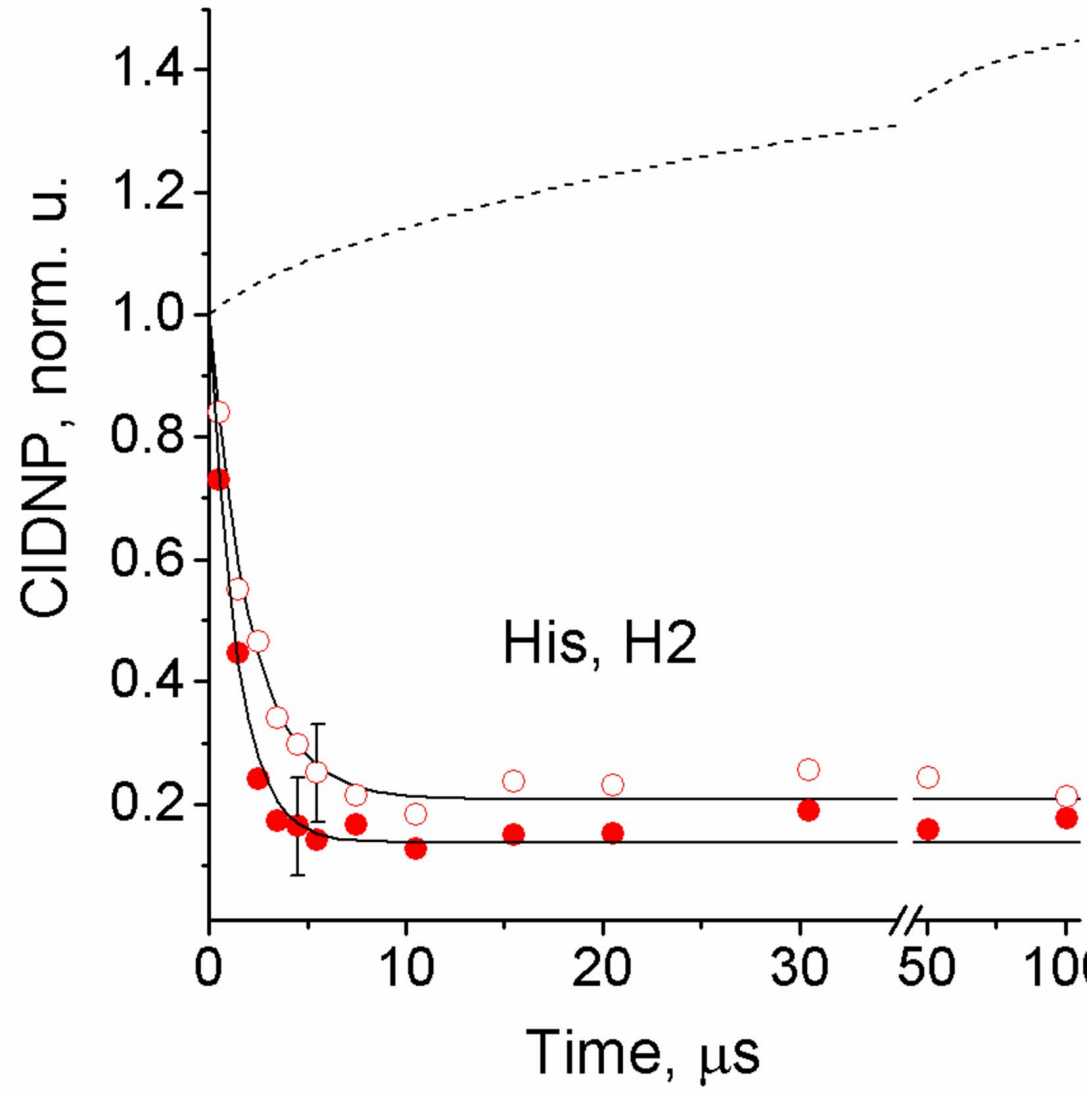
CIDNP kinetics for proton H2 of the His residue, obtained during the photoreaction between 2 mM TCBP and 20 mM (open symbols) or 40 mM (solid symbols) of the peptide His-Glu-Tyr-Gly at pH 8.8. The normalization coefficient is selected so that the first point in the calculated kinetics has an amplitude equal to 1. Dashed line – calculations with the observed rate constants of ET is equal to 0.

The CIDNP kinetic data obtained in this case for H2 of His residue are shown in Fig. 4. Measurements were performed at two peptide **1** concentrations, 20 mM and 40 mM, to reveal the contributions of intra- and intermolecular ET from Tyr residues to His radicals in the peptide **1** like in the previous study^36^. CIDNP effects in the photochemical reaction of TCBP with His and Tyr and the manifestation of the reduction reaction in the CIDNP kinetics are described in detail in our previous publications^36,39,40^. They are only briefly summarized here. In the case of the peptide **1** under study, the fast decay of the His H2 signal is a clear indication of the ET reaction from the Tyr residue to the His radical. Indeed, as a result of the nuclear spin selectivity of T_0_⟷S transitions, the nuclear spin polarization of His residue in the His^•^–Glu–TyrOH–Gly radicals which escaped the initial in-cage recombination is opposite to that in the product of the recombination reaction of the geminate RP. However, ET from Tyr residue restores the diamagnetic state of His residue in these escaped radicals, leading to cancelation of CIDNP signals of His protons which manifests in a fast CIDNP decay. Tyr radicals formed via ET carry no nuclear polarization. However, these radicals participate in F-pair processes with TCBP radicals, in which additional CIDNP is formed. An increase is actually observed in the CIDNP kinetics of Tyr protons relative to the case when there is no ET. However, this increase is less pronounced than the decay of the histidine CIDNP, which is affected by ET directly. Similarly, for TCBP radicals, in the reaction with Tyr radicals, an additional CIDNP is formed, but its sign is opposite to the polarization formed in radical reactions with His. This dependence of the CIDNP sign on the partner of the radical pair makes the CIDNP kinetics of TCBP more sensitive to ET compared to CIDNP kinetics of Tyr, and the CIDNP growth for TCBP is expressed much more strongly than for Tyr. To determine the observed rate constants of ET, CIDNP kinetics for the protons of all reaction participants were simulated (SI, Figure S3). The simulation procedure was developed and applied earlier in the study of intra- and intermolecular electron transfer from tyrosine to the histidine radical in various systems, which is described in detail in the corresponding publications^36,40^.

The ET rate constants obtained from the simulation of the CIDNP kinetics are 4.0 × 10^5^ s^− 1^ and 6.5 × 10^5^ s^− 1^ at 20 and 40 mM of His–Glu–Tyr–Gly, respectively. These observed rate constants are contributed by the rate constants of the intra- and intermolecular ET, the latter is the product of the second-order reaction rate constant and the peptide **1** concentration. Solving simple algebraic equations gives (1.5±0.5)×10^5^ s^−1^ for the rate constant of the intramolecular ET, and (1.3±0.4)×10^7^ M^−1^s^−1^ for the intermolecular ET at pH 8.8. As mentioned above, the intramolecular ET rate constant determined in the present study for peptide **1** was assigned to the reaction involving neutral tyrosine (Eq. 3), while the rate constant of intermolecular ET refers to both reaction Eqs. (4) and (5). To determine the individual rate constants of reactions (4) and (5), pH-dependence analysis is required; however, firstly, this was not the aim of the present study, and secondly, it is impossible due to the low solubility of peptide **1** in a neutral aqueous solution. The value 1.5 × 10^5^ s^−1^ is comparable to that obtained for His-Tyr peptide, 2.6 × 10^5^ s^−1^, ^36^ and is the reference for conjugate **2**.

### Electron transfer in His-Gln(BP)-Tyr-Gly conjugate 

**Fig. 5:**
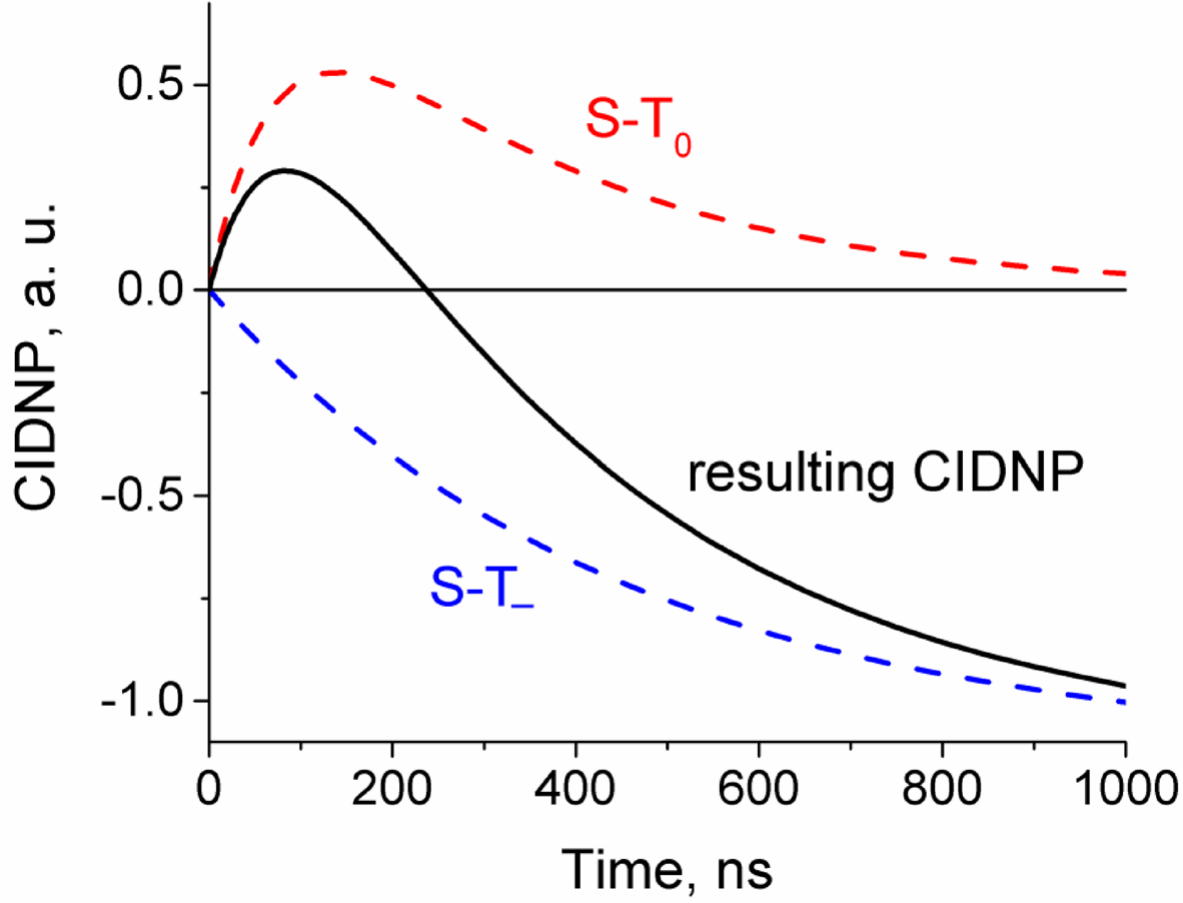
Schematic representation of CIDNP kinetics formed by S-T_0_ (upper dashed line) and S-T_−_ (lower dashed line) mechanisms in biradicals for spin 1/2 nuclei with a negative HFCC, *A* = − 0.5 mT, at a magnetic field B satisfying the inequality |*A*| < B < 2|*J*_ex_|. Solid line shows the resulting curve.

Based on previously obtained data on quenching rate constants^55^ and rate constants for the reduction of histidine radicals by tyrosine^40^, we assume that photoirradiation of such biomimetic conjugate leads to the formation of a primary spin-correlated biradical consisting of the benzophenone radical and the radical of one of the amino acids residues. The main feature of biradicals is the non-zero electronic exchange interaction 2*J*_ex_ between the radical centers throughout the lifetime of the biradical^70^. The electronic exchange interaction splits the singlet and triplet states of the biradical at zero magnetic field, where the energy gap is equal to 2|*J*_ex_|. The magnitude of the exchange interaction depends on the overlap of the unpaired electron orbitals of the radicals. A shorter distance between the radicals results in a larger 2|*J*_ex_| that suppresses the T_0_⟷S transitions at high magnetic field, while a longer distance results in a smaller electron coupling making the T_0_⟷S transitions more efficient. At high magnetic field, the Zeeman splitting becomes much larger than the average singlet-triplet splitting, B>>2|*J*_ex_|. Under these conditions, the transitions T_–_⟷S (or T_+_⟷S) are not efficient and CIDNP effect is formed in T_0_⟷S transitions according to radical-pair mechanism^71–73^. Due to the impossibility of the radical centers separation, the biradicals can only recombine generating geminate CIDNP. The magnetic interactions of the electrons and nuclei in the biradicals, namely the difference in their g-factors and the hyperfine interaction between the unpaired electron and the nuclei, drive the transition from the T_0_ to the S state. The rates of these transitions are not the same for the different configurations of the nuclear spins. In this way, all the biradicals produced are divided into sub-ensembles: those with nuclear spin states for which the rate of transition from T_0_ to S is higher, and those with nuclear spin states with lower rate. Thus, the CIDNP kinetics show an initial increase, followed by a maximum and then a decay to zero (upper dashed line in Figure 5)^74^. According to the radical-pair T_0_⟷S (or S⟷T_0_, which is fully equivalent) mechanism, CIDNP spectra of biradicals recorded at high magnetic field show the NMR signals of emission and enhanced absorption, by contrast to low field, where emission effects observed in the case of triplet-born biradicals with negative *J*_ex_ due to T_–_⟷S transitions.

### TR CIDNP in His-Gln(BP)-Tyr-Gly conjugate

**Fig. 6:**
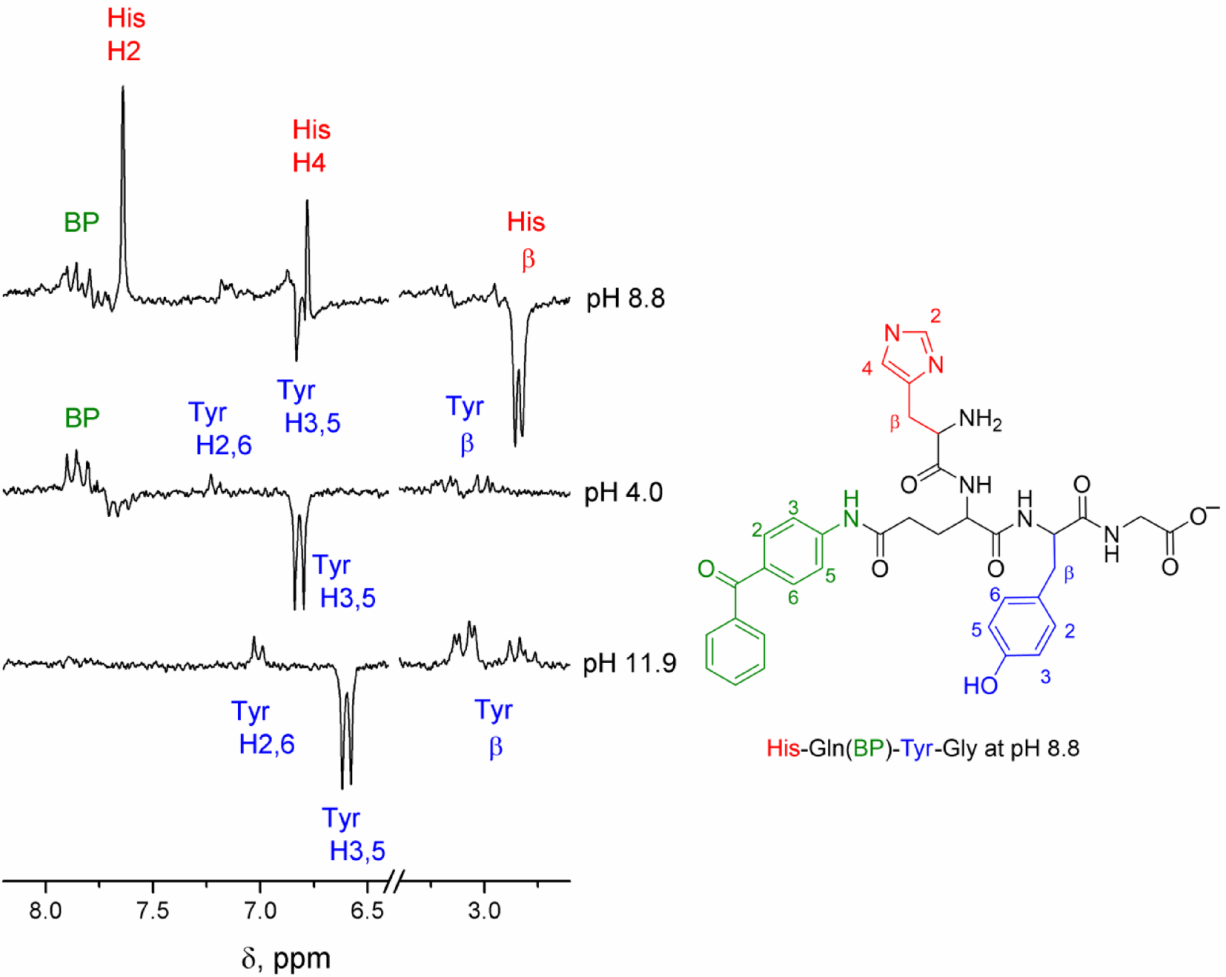
CIDNP spectra of 0.3 mM His-Gln(BP)-Tyr-Gly at pH 8.8, 4.0, 11.9 obtained just after the laser pulse. The number of scans was equal to 64. The detecting radiofrequency pulse (RF-pulse) was 5 µs.

**Fig. 7:**
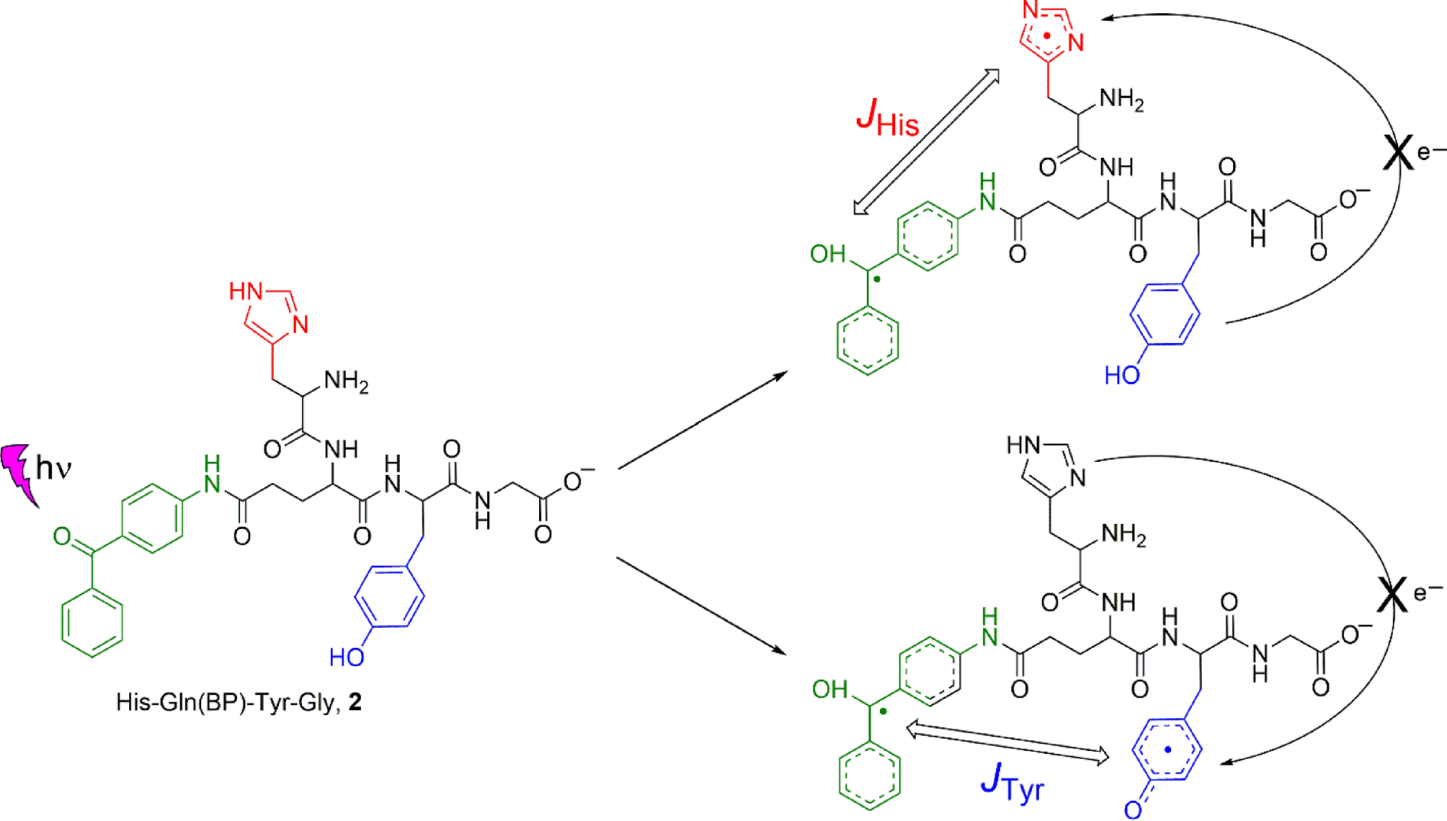
Two types of His-Gln(BP)-Tyr-Gly biradicals formed under photooxidation at slightly basic solution. There was no intra- or intermolecular electron transfer from Tyr to His^•^ detected. Exchange interaction in two types of His-Gln(BP)-Tyr-Gly biradicals is designated: *J*_His_ and *J*_Tyr_.

Representative CIDNP spectra obtained after photoirradiation of the His-Gln(BP)-Tyr-Gly conjugate at pH 4.0, 8.8 and 11.9 are shown in Fig. 6. The spectra were obtained without delay after the laser pulse using detecting RF-pulse of 5 µs and contain signals of products formed as a result of the recombination reaction. CIDNP for tyrosine protons was observed at all pH values, and for histidine protons – in the pH range from 8.7 to 10.8. These observations correspond to changes in the relative reactivity of amino acid residues: histidine with protonated imidazole (pH 4) is less reactive than tyrosine; histidine with neutral imidazole has reactivity comparable to that of tyrosine (neutral to slightly basic pH); tyrosinate anion residue is more reactive than histidine one (pH 11.9). Thus, when the photoreaction is carried out at pH 8.8, two types of biradicals are formed: the first − consisting of benzophenone and tyrosine radicals, the second − consisting of benzophenone and histidine radicals (Fig. 7). At pH 4.0 and 11.9 only one type of biradical is formed − with radical centers on benzophenone and tyrosine moieties. The signs of the CIDNP signals are in accordance with Kaptein’s rule for triplet precursor multiplicity^63^, with the known signs of the HFCCs for polarized protons, and a g-factor of the benzophenone radical greater^64^ than that of the histidine radical^65^ and less than that of the tyrosine radical^66^.

**Fig. 8:**
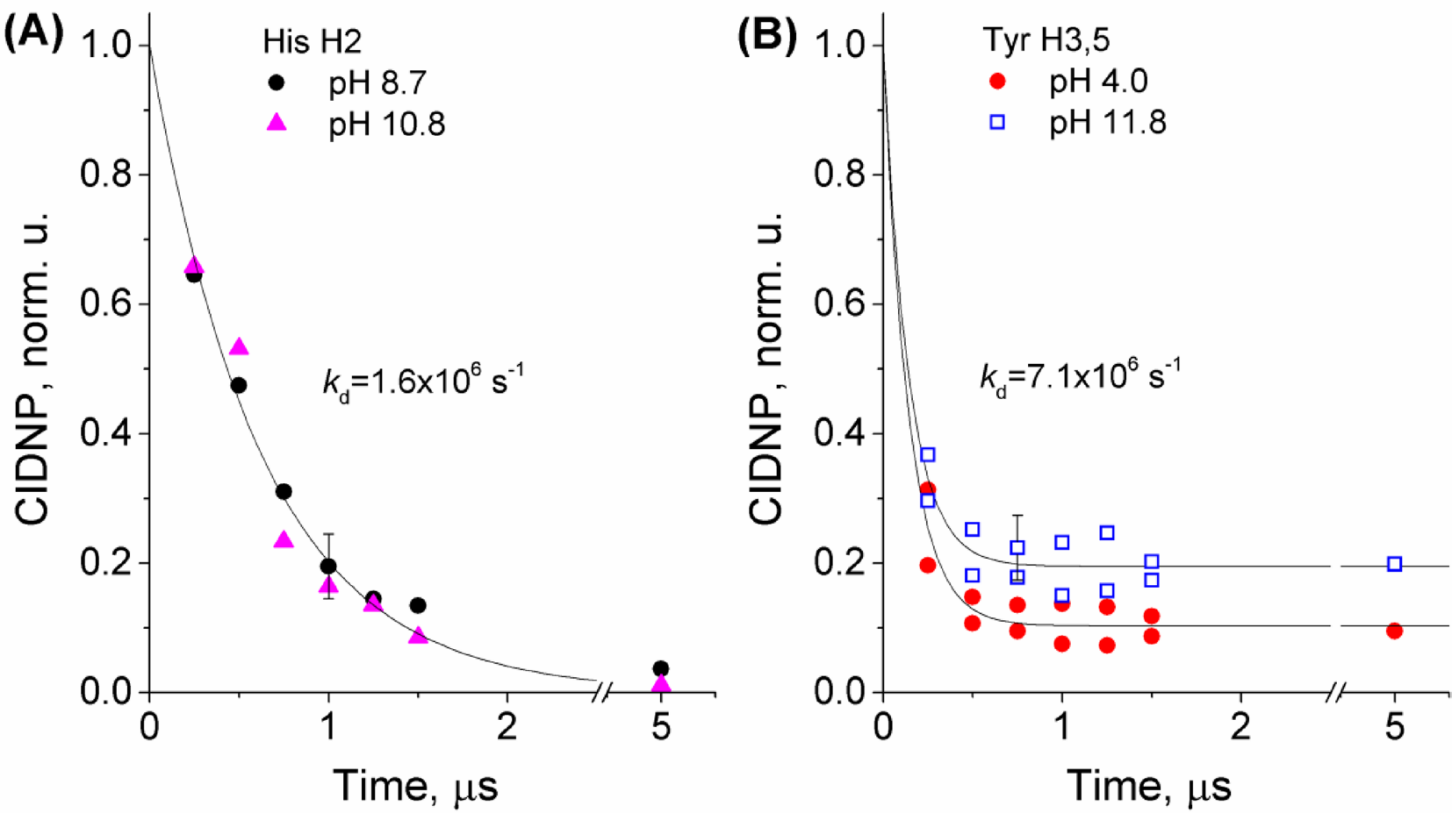
CIDNP kinetics obtained during irradiation of 0.3 mM His-Gln(BP)-Tyr-Gly for (A) H2 of His residue at pH 8.7 (circles) and 10.8 (triangles); (B) H3,5 of Tyr residue at pH 4.0 (circles) and 11.8 (squares). Detecting RF-pulse was 0.5 µs. Number of scans 128.

In order to answer the question of whether intramolecular electron transfer from tyrosine residue to histidine radical takes place, CIDNP kinetic data for H2 of His residue in the pH range from 8.7 to 10.8 were analyzed. It turned out that the kinetics are pH-independent in this range, the data corresponding to the utmost values are shown in Fig. 8 (A).

Typically, the geminate evolution of the biradical spin states takes hundreds of nanoseconds^75^. In contrast to peptide **1**, all reaction products of conjugate **2** photoirradiation are in-cage, as there is no diffusion separation of the biradicals. In general, at high magnetic field, S⟷T_0_ mixing is the dominant pathway of triplet-to-singlet interconversion^75^. The triplet biradicals resulting from the photochemical reaction can be divided into sub-ensembles with respect to nuclear spin orientation. These sub-ensembles have different rates of singlet-triplet conversion: the faster S⟷T_0_ transitions are followed by slower ones, and eventually all sub-ensembles of the T_0_ term acquire singlet character and yield diamagnetic products. Accordingly, the CIDNP passes through a maximum and goes to zero with time (Fig. 5)^75^. However, we were not able to use detection RF pulses shorter than 0.5 µs, so our time resolution did not allow to detect the initial CIDNP growth. The finite RF pulse duration is taken into account by defining the points on the time scale that correspond to the time between the laser pulse and the center of RF-pulse, which previous studies have shown to be a good approximation. Therefore, the time in Fig. 8 is shifted relative to the delay *τ* between the laser pulse and the RF pulse by half the length of the latter: in Fig. 8*τ* = 0 µs corresponds to the time 0.25 µs, *τ* = 0.25 µs − to the time 0.5 µs, etc.

At high magnetic field, S⟷T_0_ transitions caused by g-factor difference of the radical centers occur on a nanosecond time scale (~ 15 ns at B = 4.7 T and Δg ~ 10^− 3^). On the same time scale, the formation and decay of CIDNP in spin-correlated radical pairs occurs. The limiting stage that determines the lifetime of triplet biradicals is the time of electron dipole-dipole relaxation from the T_±_ states to the T_0_ state, depleted as a result of interconversion to S state and recombination. Spin evolution of repopulated T_0_ state leads to CIDNP formation according to S⟷T_0_ mechanism. Thus, the decay of CIDNP kinetics in Fig. 8 (A) is determined by dipole-dipole relaxation, and the characteristic decay rate constant *k*_d_=1.6 × 10^6^ s^− 1^ can be roughly attributed to the inverse relaxation time. Since the characteristic decay time is an order of magnitude greater than the assumed intramolecular ET rate constant, the latter has no chance of manifesting itself during the lifetime of the biradical.

The coincidence of kinetics at pH 8.8 and 10.8 indicates that there is no acceleration of CIDNP decay upon deprotonation of tyrosine with increasing pH. This means that during the biradical lifetime, there is no intermolecular electron transfer from the tyrosinate anion at the conjugate concentration of 0.3 mM, while the absence of intramolecular ET from tyrosinate anion is in accordance with the previous investigation^36^.

CIDNP kinetics for H3,5 protons of the Tyr residue were obtained during irradiation of 0.3 mM His-Gln(BP)-Tyr-Gly in the acidic (pH 4.0) and basic (pH 11.9) conditions (see Fig. 8, B). The decay was much faster than that of His protons and is essentially represented by only the first point in the CIDNP kinetics. The decay rate constant *k*_d_ is estimated at 7.1 × 10^6^ s^− 1^. In contrast to the case of His, a non-zero stationary CIDNP is observed. In the case of CIDNP formed in the reaction of free radicals, non-zero stationary CIDNP is determined by nuclear paramagnetic relaxation in the radicals caused by the anisotropy of hyperfine interaction, and the latter determines the characteristic relaxation time of 10–1000 µs. In the present case, non-zero stationary CIDNP may be caused by nuclear relaxation in biradical. So, it was revealed that two types of His-Gln(BP)-Tyr-Gly biradicals formed under photooxidation differ in their characteristics, in particular in the dipole-dipole relaxation time.

Thus, time-resolved CIDNP experiments on the synthetic peptide His-Glu-Tyr-Gly and the conjugate His-Gln(BP)-Tyr-Gly identified intramolecular and intermolecular electron transfer. However, to characterize the magnetic interactions influencing ET processes, field-dependent CIDNP studies are necessary. Therefore, in the next part of the article we describe the results of the experiments with the conjugate His-Gln(BP)-Tyr-Gly that determined a range of magnetic fields where the exchange interaction matches the electronic Zeeman interaction. The obtained CIDNP field dependences allowed to prove the biradical nature of the short-lived RP intermediate of the photochemical reaction, and to characterize the biradical geometry in acidic and basic conditions. In addition, the numerical modelling of CIDNP field dependences using the DFT calculated HFCCs allowed to extract the values the exchange interaction. This integrated approach demonstrates the effectiveness of combining time-resolved and field-dependent CIDNP methods to probe fast ET processes in biomimetic systems.

### Field-dependent CIDNP in His-Gln(BP)-Tyr-Gly conjugate

**Fig. 9:**
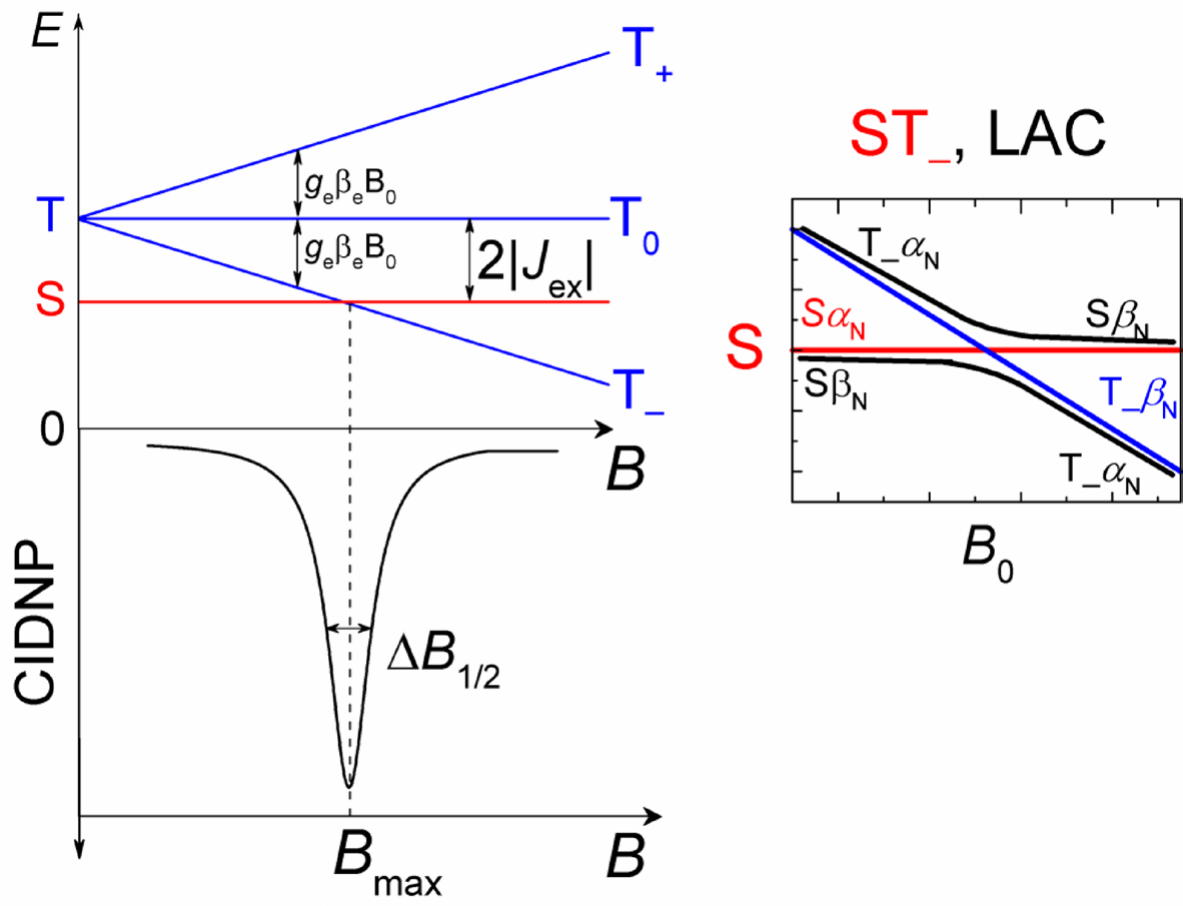
Energy level diagram of a biradical with negative exchange interaction at external magnetic field *B*. The region of S and T_−_ level crossing (LC) is magnified in the insert. It shows that LC is turned to the level anti-crossing (LAC) by hyperfine interaction at *B*_max_ where the coherent electron and nuclear spin flip-flop process |T_–_α_N_> ⟷|Sβ_N_ > takes place, explaining the dependence of CIDNP on the magnetic field.

The efficiency of triplet-singlet transition, and therefore photo-CIDNP formation in biradicals is highly dependent on magnetic field strength. When an external magnetic field is applied, the triplet state is split by the Zeeman interaction into T_+_, T_0_ and T_−_ sublevels. The singlet and one of the triplet sublevels cross at the magnetic field B = 2|*J*_ex_|. Two regimes (cases) of magnetic field influence can be distinguished: (1) when the external magnetic field is comparable to 2|*J*_ex_|, we will call it the low-field case, and (2) when the external magnetic field is much larger than 2|*J*_ex_|, we will call it the high-field case. If we look at biradicals with a long carbon chain (e.g. eight methylene groups and more connecting the radical centers), such as the conjugate His-Gln(BP)-Tyr-Gly studied in this work, then comparatively small negative values of the electronic exchange interaction are characteristic for them: |*J*_ex_|<0.1 T, *J*_ex_ <0. When *J*_ex_ is negative, at zero magnetic field the singlet electronic state S is below the triplet manifold, and at magnetic field strength equal to 2|*J*_ex_| there is an S-T_–_ electron energy level crossing (Fig. 9). In the presence of isotropic hyperfine interaction (HFI) with magnetic nuclei, the level crossing (LC) turns into a level anti-crossing (LAC) for the Sβ_N_ and T_–_α_N_ levels of the biradical (subscript “N” denotes the nuclear spin state); the Sα_N_ and T_–_β_N_ levels are not mixed by the hyperfine interaction. The HFI in the vicinity of LAC induces coherent electron-nuclear spin transitions |T_–_α_N_>⟷|Sβ_N_ > with the conservation of the z-projection of total electron and nuclear spin. Therefore, the spin mixing at the LAC leads to a predominance of the β_N_ nuclear spin state in the recombination product formed from singlet electronic state and thus to the formation of emissive CIDNP in diamagnetic product. This is shown in the bottom plot as the Lorentz-peak shaped line in Figure 9.

**Fig. 10:**
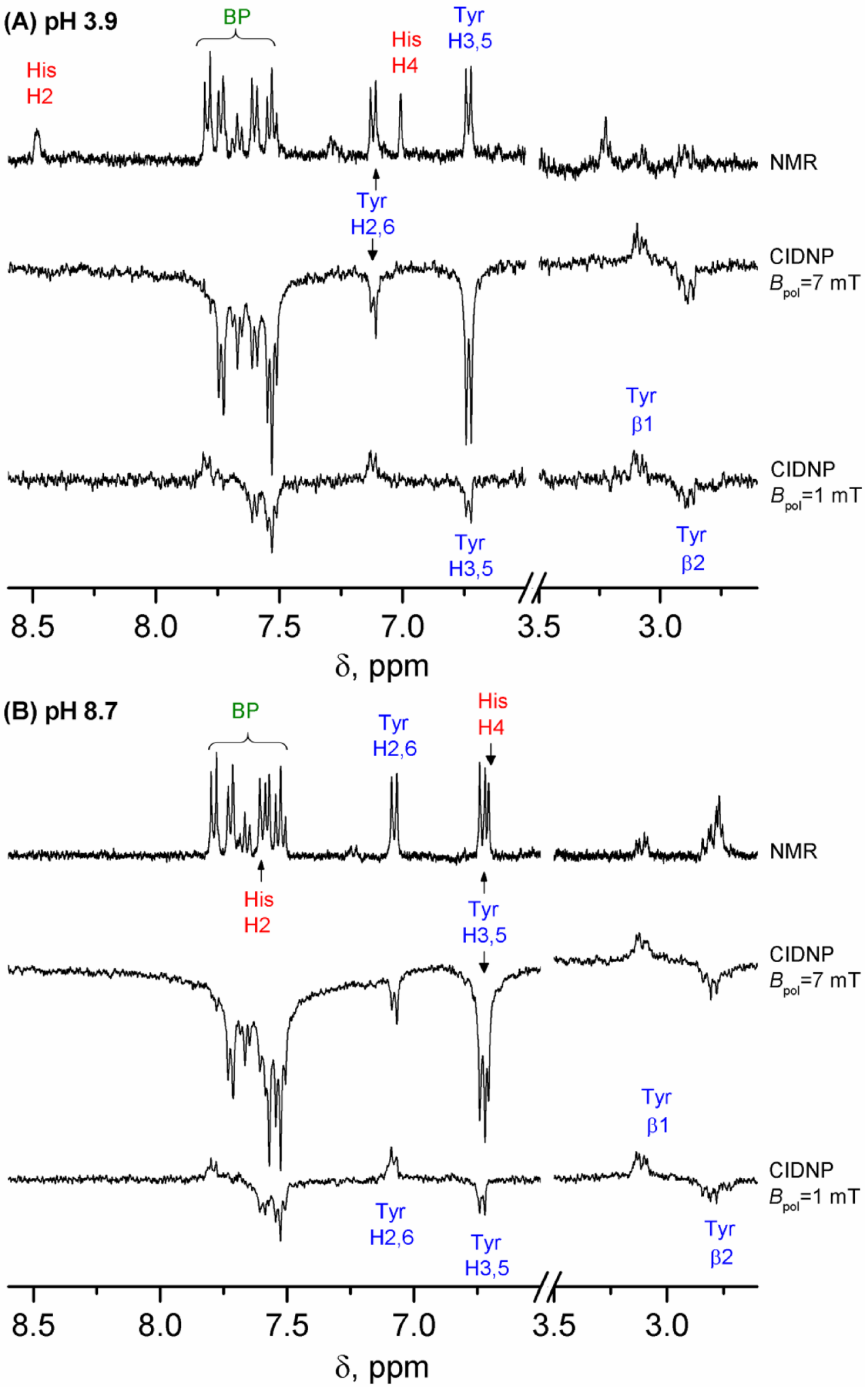
(A, B) (top) 400 MHz ^1^H NMR spectrum (32 scans), obtained for the 0.12 mM solution of His-Gln(BP)-Tyr-Gly in D_2_O. (middle and bottom) 400 MHz ^1^H CIDNP spectra (16 scans), taken after the irradiation of the 0.12 mM solution of His-Gln(BP)-Tyr-Gly in D_2_O at *B*_pol_ = 7 mT (middle) or *B*_pol_ = 1 mT (bottom). The optical density was 1.4 cm^− 1^ at 265 nm. The irradiation time was 3 s per scan. The temperature was 25 °C. The pH values of aqueous solution were 3.9 (A), and 8.7 (B).

Magnetic field dependence of CIDNP allows us to reveal exchange interaction in short-lived biradicals formed in photoinduced intramolecular ET reaction in conjugate His-Gln(BP)-Tyr-Gly. The magnitude of CIDNP effect formed via *J*-resonance mechanism at low magnetic fields is significantly higher than that formed via S⟷T_0_ mechanism at high fields. Typical NMR spectra obtained before irradiation and CIDNP spectra of the His-Gln(BP)-Tyr-Gly conjugate at magnetic field 1 mT and 7 mT at pH 3.9 and 8.7 of the aqueous solution are shown in Fig. 10 (CIDNP spectrum at pH 11.8 is given in Figure S4 in Supporting information). In the CIDNP spectra obtained at 7 mT, the same protons are polarized as at high magnetic field, but all the signals are emissive. This is in agreement with the *J*-resonance mechanism of CIDNP generation at low field:^70^ the CIDNP sign does not depend on the sign of the HFCC, but depends on the product of the sign of the exchange interaction *J*_*ex*_ and the multiplicity γ of the precursor (γ > 0 for triplet precursor and γ < 0 for singlet precursor). The emission sign of the signals corresponds to the T_–_⟷S mechanism of CIDNP formation. However, stationary CIDNP spectra provide only qualitative information about the distribution of the HFCCs in the products, since the absolute and relative line intensities depend strongly on the experimental conditions and the relationship between irradiation time, sample transfer time, and the relaxation time of the different nuclei.

At magnetic field comparable to the typical value of proton HFCC (about 1 mT and below), no net polarization is expected because there is no preferred direction in space with respect to hyperfine interaction. However, in the case of two or more spins, a strong spin order is formed at zero field because the nuclear spin states are selected with respect to their total momentum^76,77^. This results in an unusual spectral pattern observed after the system is brought to a high detection field (see CIDNP spectra obtained at 1 mT in Fig. 10). Moreover, in this situation the singlet spin order of methylene protons is formed and hyperpolarization in diamagnetic product can be long-lived, relaxing much slower than the longitudinal magnetization^78^. The reason is that dipolar relaxation (the dominant relaxation mechanism for protons) cannot drive singlet-triplet transitions; consequently, singlet-triplet relaxation is caused by less efficient mechanisms and slows down. For the β protons of amino acids, the strong spin order is formed at 1 T and below. Therefore, for the β protons at the magnetic field of 7 mT, absorption/emission NMR signals are observed in addition to the net emission signal.

**Fig. 11:**
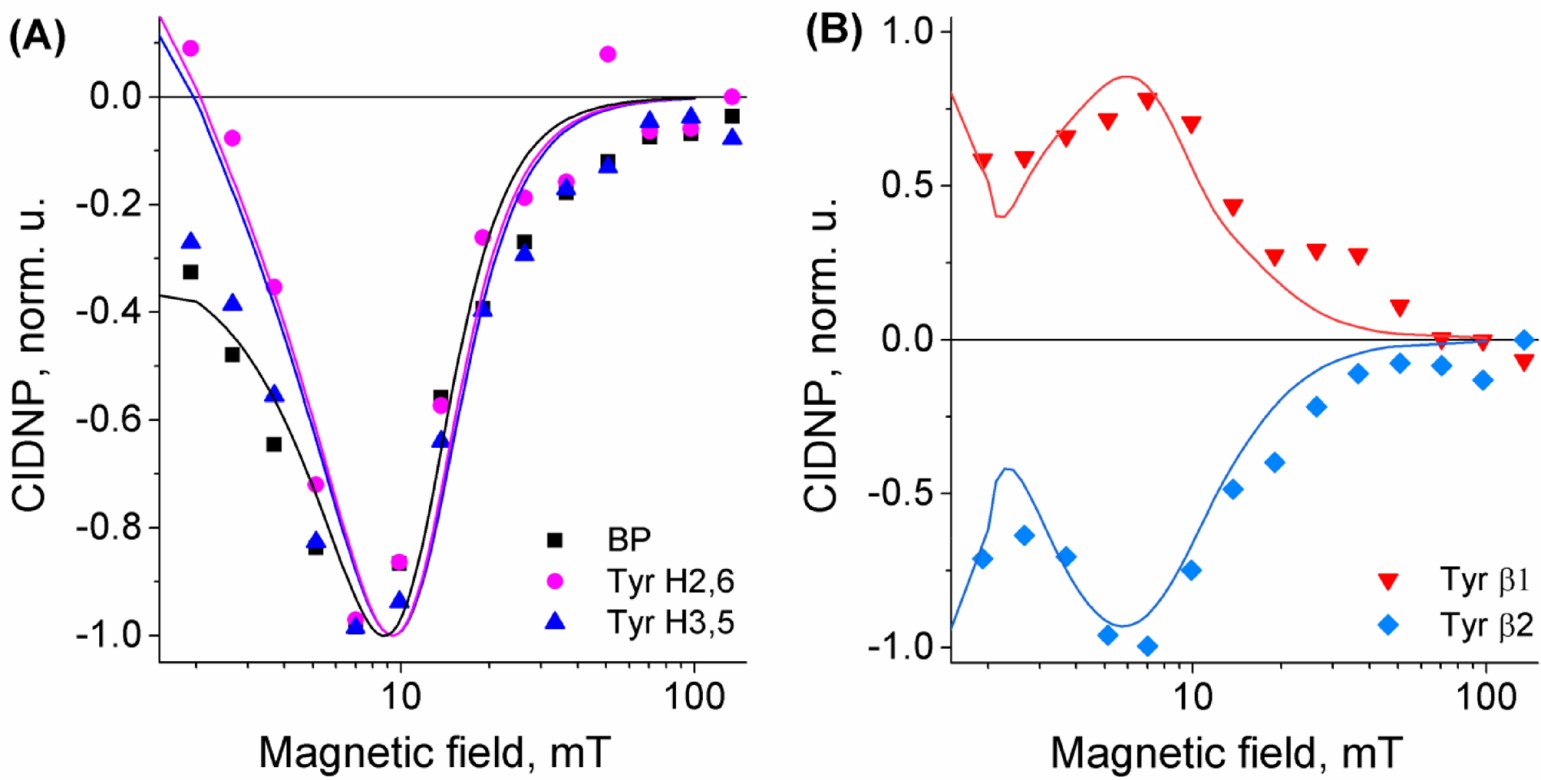
Experimental ^1^H CIDNP field dependencies for BP and Tyr protons obtained in photoreactions of 0.12 mM of His-Gln(BP)-Tyr-Gly in D_2_O at pH 3.9. The zooming factor of the vertical axis in (B) is multiplied by 7.5. Lines show the result of CIDNP calculation with parameters listed in Table S1 in Supporting information.

The field dependence of CIDNP for His-Gln(BP)-Tyr-Gly protons at pH 3.9 in aqueous solution is shown in Fig. 11, two other cases for pH 8.7 and 11.8 are shown in Figure S5 in SI. At pH 3.9 only polarized signals from tyrosine and benzophenone residue protons are observed, since histidine with protonated imidazole is less reactive than tyrosine. At pH 8.7, histidine with deprotonated imidazole exhibits a reactivity comparable to tyrosine, and the observed histidine residue signals overlap with the comparable in size signals of tyrosine and benzophenone residues. At pH 11.7, mainly the signals of tyrosine and benzophenone are observed, which indicates the increased reactivity of tyrosinate anion towards benzophenone triplets.

The observed CIDNP magnetic field dependencies have the same shape for all polarized protons at each pH studied, with almost coinciding maxima (*B*_max_) but slightly different width at half height (*w*_1/2_). The presence and coincidence of emission maxima for all protons can readily be accounted for T_–_⟷S mechanism of CIDNP formation. The T_–_⟷S intersystem crossing involves simultaneous flip of electron and one of the nuclear spins from α to β spin states (|T_–_α_N_ > states transfer to |Sβ_N_ > irrespective of the sign of HFI). The field dependence of CIDNP is determined by the magneto-resonance parameters (exchange and hyperfine interactions) of intermediate biradicals^47,70,79^.

To get more detailed information about the exchange interaction in the biradical of conjugate His-Gln(BP)-Tyr-Gly, we simulated the CIDNP field dependence obtained at pH 3.9 using the numerical approach published by some of us recently^80^. The simulation is shown by a solid line in the Fig. 11. Simulation parameters are listed in Table S1 in SI. Further data supporting the use of the simulation parameters values are found in Tables S2-S3 and Figure S6 in SI.

**Fig. 12:**
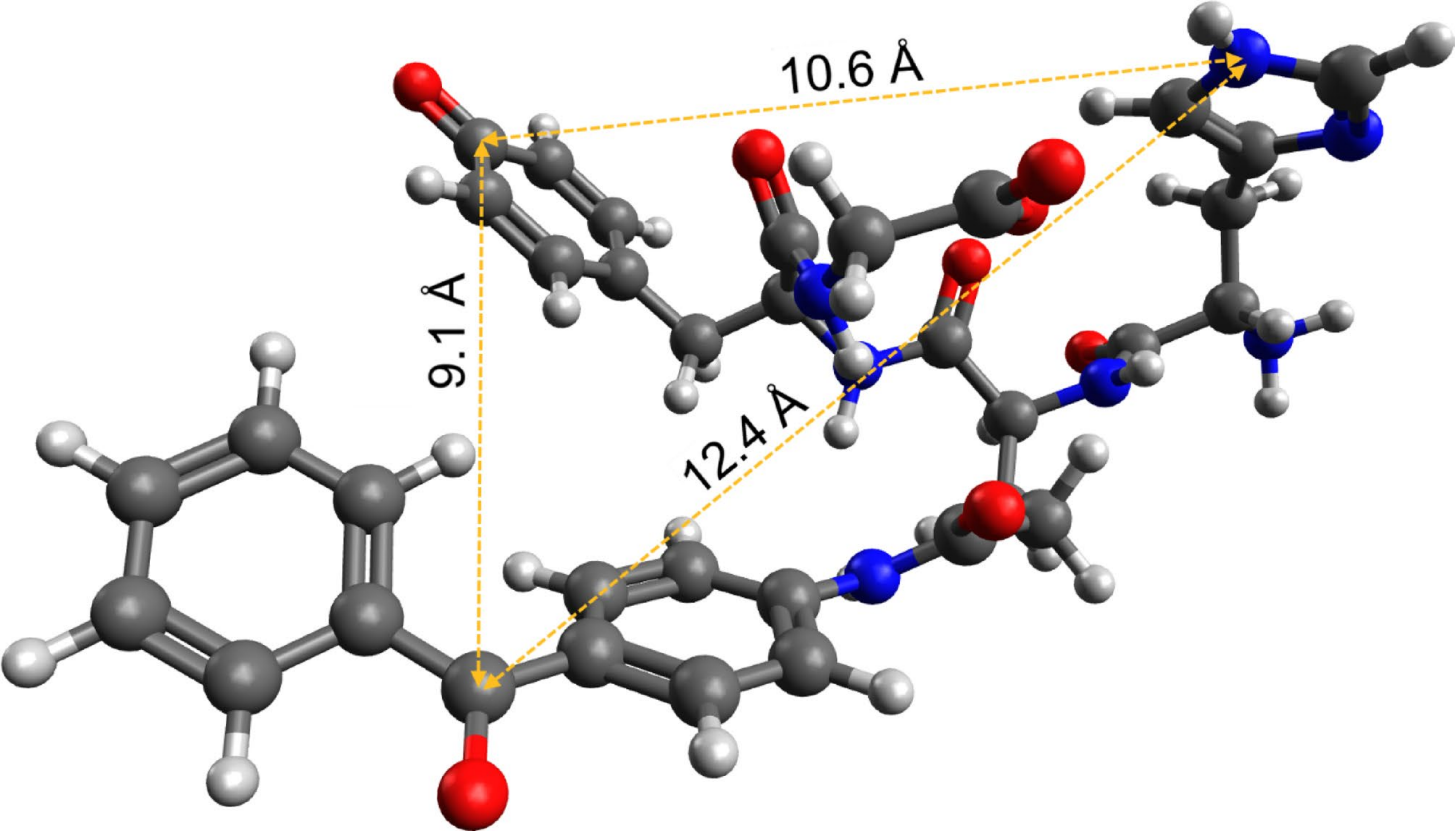
Optimized geometry of biradical in its triplet state formed from His-Gln(BP)-Tyr-Gly conjugate with neutral amino group of His. Electron density is located on BP^•^ and Tyr^•^ radical moieties, no spin density found on His residue. The calculation was done using TPSS/def2-SVP method accounting for the solvent (water) via the SMD model. Distances between centers are: His and Tyr residues – 10.6 Å; BP and His – 12.4 Å; BP and Tyr – 9.1 Å.

From fitting the CIDNP data we were able to reveal a non-zero exchange coupling 2*J*_ex_ = −8.78 mT in biradical of conjugate His-Gln(BP)-Tyr-Gly at acidic pH. It is smaller and opposite in sign to values 2*J*_ex_ = 17–30 mT obtained using the same procedure for a series of donor-bridge-acceptor compounds D-X-A studied by some of us earlier^53,79^. The TPSS/def2-SVP^81,82^ method accounting for the solvent (water) via the SMD^83^ model was used to optimize the geometries of biradical in its triplet state formed from His-Gln(BP)-Tyr-Gly conjugate (Fig. 12), see Materials and Methods section for details. The distance 9.1 Å between the Tyr and BP residues is much shorter than about 20 Å in biradical of the donor-bridge-acceptor compounds D-X-A^79^. However, the exchange interaction is smaller, which could be explained by two reasons. The first one is that positive *J*_ex_ of D-X-A biradical indicates the involvement of the mediating solvent molecular orbital into the electron-electron coupling, extending its spatial range. The second one stems from the results of geometry optimization where the aromatic rings of BP and Tyr lie nearly in the same plane, thus suppressing the p-orbital overlap.

**Fig. 13:**
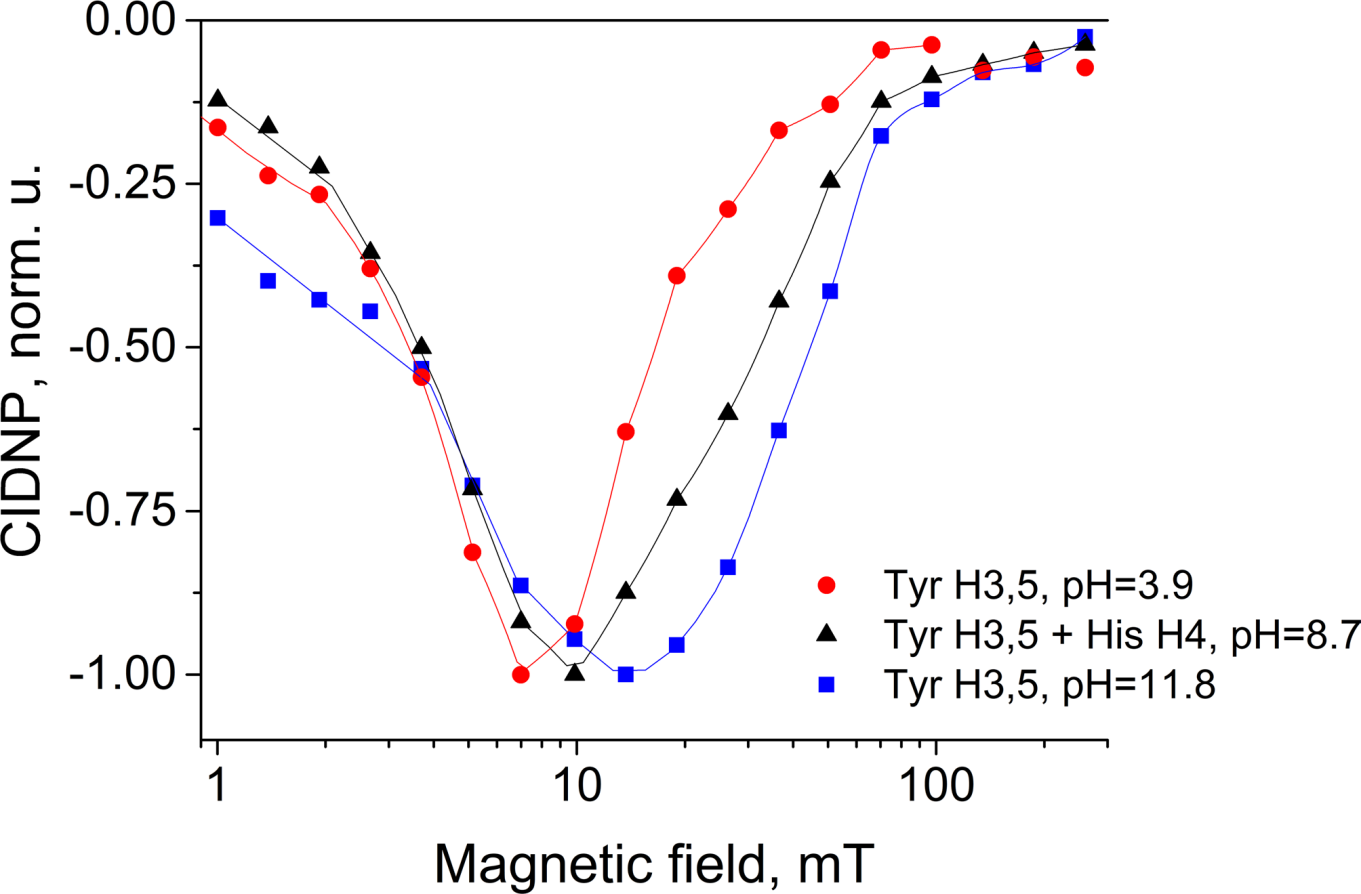
^1^H CIDNP field dependences for Tyr H3,5 (pH 3.9 and 11.7) and sum of Tyr H3,5 and His H4 (pH 8.7) protons obtained in the photoreactions of 0.12 mM of His-Gln(BP)-Tyr-Gly in D_2_O. The lines are guides for the eye.

The value of 2*J*_ex_ is a sensitive probe of the conformation of a short-lived biradical, reflecting the conformational ensemble of the precursor diamagnetic conjugate His-Gln(BP)-Tyr-Gly in solution. A comparison of the field dependences obtained at pH values of 3.9, 8.7 and 11.9 is shown in Fig. 13. Simulation of the field dependences measured at pH values 8.7 and 11.9 is underway and will be published elsewhere. It is seen that the half-width of the *J*-resonance curve increases with the increasing pH: *B*_max_ is ~ 7 mT at pH 3.9, ~ 10 mT at pH 8.7, and ~ 14 mT at pH 11.8. It is known that the greater the exchange interaction is, the larger is the overlap of the orbitals occupied by the unpaired electrons. This could be either due to the closer proximity or more favorable mutual orientation of the radicals. In basic solution, His-Gln(BP)-Tyr-Gly contains tyrosine in the form of its anion, in acidic and neutral solution – in neutral form. However, at all pH values, the oxidized form of tyrosine is its neutral radical TyrO^•^, since its conjugated acid – cation radical TyrOH^•+^ with p*K*_a_< (−1) quickly deprotonates. Thus, the increase in exchange interaction in basic solution is apparently associated with a change in the structure of the benzophenone radical - from ketyl to anion-radical (p*K*_a_ of ketyl radical of benzophenone is 9.4)^84^.

## Conclusion

This study provides a comparative analysis of photoinduced electron transfer in two model systems, the tetra-peptide His-Glu-Tyr-Gly and the conjugate His-Gln(BP)-Tyr-Gly (containing benzophenone BP), using chemically induced dynamic nuclear polarization. The findings reveal distinct differences in the mechanisms and kinetics of electron transfer processes in these systems, with an emphasis on the effects of molecular structure and pH on electron transfer dynamics. His-Glu-Tyr-Gly was studied by time-resolved CIDNP method in photosensitized reactions with 3,3’,4,4’-tetracarboxy benzophenone. At neutral and slightly basic pH, two types of radical pairs were detected due to the similar reactivity of histidine and tyrosine with triplet-excited TCBP under these conditions. Additionally, electron transfer from tyrosine to histidine radicals was observed. Analysis of the CIDNP kinetics demonstrated clear contributions from both intra- and intermolecular electron transfer pathways, and its rate constants were determined.

For the conjugate His-Gln(BP)-Tyr-Gly the kinetics and magnetic field dependence of ^1^H CIDNP were measured and analyzed at variable pH to reveal the influence of protonation states of reactants on photoinduced electron transfer. His-Gln(BP)-Tyr-Gly showed, like the peptide His-Glu-Tyr-Gly a complex pH-dependent behavior with respect to the electron transfer reaction. At acidic pH, electron transfer predominantly involved tyrosine, resulting in biradicals formed by benzophenone and tyrosine radicals. At neutral and slightly basic pH, both histidine and tyrosine participated in electron transfer, forming biradicals with distinct geometries but similar exchange interactions. At strongly basic pH, tyrosine in its deprotonated form (tyrosinate) dominated the electron transfer process. Magnetic field dependent CIDNP data revealed nonzero exchange interactions, indicating close spatial proximity of the radical centers of 4-amino benzophenone and Tyr residues. No electron transfer from tyrosine to histidine radicals was observed in the oxidized conjugate His-Gln(BP)-Tyr-Gly under any condition because of its short lifetime, contrasting with peptide His-Glu-Tyr-Gly. This study demonstrates how pH modulates electron transfer pathways and biradical characteristics in the conjugate His-Gln(BP)-Tyr-Gly, providing information into the interplay between protonation states and electron transfer dynamics. These findings underscore the utility of CIDNP in investigating complex processes including the formation of transient paramagnetic species in model systems and contribute to understanding photoinduced ET in biologically relevant contexts.

The methodological advancements in this study, based on the combination of the time-resolved and the magnetic field dependent CIDNP techniques provide a robust framework for electron transfer studies with atomic resolution. The replacement of high-power lasers with LEDs enhanced accessibility, portability, and safety, allowing rapid and stable experimentation. Usually, biochemists work with the low sample concentration (≤ 1µM) that typically require photosensitizers with long triplet-state lifetimes to ensure radical pair formation within the triplet lifetime. However, when the photosensitizer and quencher are linked and in close proximity, the triplet lifetime can be shorter, as exemplified by benzophenone (1.5 µs)^85^. This highlights the potential for designing compact and efficient electron transfer systems where close spatial arrangement compensates for shorter triplet-state lifetimes.

## Materials and methods

### Chemicals

The synthesis of the His-Glu-Tyr-Gly peptide and His-Gln(BP)-Tyr-Gly conjugate (Fig. 1) is described in Supporting information. The molecular structures as well as HPLC, ESI-MS and proton NMR spectra of the intermediate and final products of the synthesis are shown in Figures S7-S15 in the supporting information. Additional information is presented in Tables S4-S5. All chemicals for the synthesis were purchased from Sigma-Aldrich, Carl Roth GmbH, Iris, Acros, VWR and ABCR and if not explicitly mentioned used without any purification. The following chemicals were obtained from Sigma-Aldrich: DCl (35 wt% solution in D_2_O, 99 + atom % D), NaOD (40 wt% solution in D_2_O, 99 + atom % D), and D_2_O (99.9%).

### Acidity of solutions and acidity constants of reactants in D_2_O

The ^1^H CIDNP measurements were performed in D_2_O. The acidity of the NMR samples was adjusted by the addition of small quantities of DCl or NaOD. The acidity of the D_2_O solutions was measured using a pH meter calibrated in H_2_O. The pH readings and p*K*_a_ values obtained in this way correspond to what are known as pH^*^ and p*K*_a_^*^, respectively, in accordance with the following formula: p*K*_a_=0.929p*K*_a_^*^+0.42^86^. Determined in this way was p*K*_a_^*^ of phenol of tyrosine residue in conjugate **2** was 10.8 (see Figure S2 in SI). This gives p*K*_a_=10.4. However, for convenience, we show the exchangeable protons rather than deuterium atoms, and use the terms pH and p*K*_a_. Therefore, pH 10.8 here gives the conjugate with half neutral tyrosine and half tyrosinate anion.

### Time-resolved CIDNP

Our setup for TR-CIDNP measurements has already been described in detail^41^. The samples purged with pure nitrogen gas and sealed in a standard NMR Pyrex ampule were irradiated in the probe of a Bruker DPX-200 NMR spectrometer (magnetic field 4.7 Т, resonance frequency of protons 200 MHz) by laser pulses from a COMPEX Lambda Physik XeCl excimer laser (308 nm, 15 ns pulse duration, pulse energy ~ 70 mJ). Light to the sample was guided using an optical system with a prism and a light-guide quartz rod (diameter 5 mm). The TR-CIDNP spectra were obtained in the following way: (1) saturation broadband radio frequency (RF) pulses, (2) a 15 ns laser pulse triggered by the spectrometer and (3) a detecting RF pulse with duration of 1 µs (under TR–CIDNP study of His-Glu-Tyr-Gly peptide) or 5 µs (to register CIDNP spectra for His-Gln(BP)-Tyr-Gly conjugate, shown in Fig. 6), or 0.5 µs (to study the CIDNP kinetics for His-Gln(BP)-Tyr-Gly conjugate, shown in Fig. 8) followed by FID acquisition. The laser pulse was synchronized with the front edge of the RF pulse. As the background signals from Boltzmann polarization were suppressed by saturation pulses, in the CIDNP spectra only the NMR signals from the polarized products of the cyclic photochemical reaction appear.

### Field-cycling NMR and CIDNP

The experimental setup allowing fast field-cycling NMR experiments over a wide range of magnetic fields is described in detail in ref^87^. The setup allows the detection of high resolution NMR spectra at *B*_0_ = 9.4 T and light irradiation of the NMR sample^53,54^ at any desired magnetic field strength *B*_pol_ between 1 mT and 9.4 T by the precise positioning of the sample in the stray field of the spectrometer cryomagnet. The field resolution is determined by the field gradient across the illuminated sample volume. A ^1^H CIDNP experiment with mechanical field cycling is composed of five consecutive steps: (1) thermal polarization suppression at high magnetic field by application of a series 90° RF-pulses followed by a pulsed *B*_*z*_-field gradient; (2) transfer of the sample to field *B*_pol_; (3) CIDNP generation by light irradiation at the desired field *B*_pol_; (4) transfer of the polarized reaction products to the observation field *B*_0_ = 9.4 T of the NMR spectrometer; (5) measurement of the NMR signal at *B*_0_ = 9.4 T. During step 3, the sample is irradiated for 3 s by a 265 nm 1 W LED light source via a quartz light guide sealed to it.

### Simulation of magnetic field dependence of CIDNP

The theoretical model used for CIDNP simulation was as following. The Hamiltonian *Ĥ* comprises the Zeeman interaction of the electron spins, the isotropic hyperfine interaction, and the exchange interaction. Fixed average value exchange interaction 2*J*_ex_ was used, and the effects of *J*_ex_ modulation due to the relative mobility of the radicals was modeled by the amplitude of *R*_*Jex*_ parametrizing the dephasing rate of T⟷S coherences *W*_*deph*_*~1/2∙(R*_*Jex*_*)*^*2*^*∙τ*_*Jex*_, *τ*_*Jex*_ = 10^−12^ s. Longitudinal relaxation of an electron spin was described using simple Bloch model by a field independent relaxation time *T*_*1e*_. Values of 2*J*_ex_, *R*_*Jex*_, and *T*_*1e*_ were optimized to give the best fit of the experimental Tyr H2,6 and H3,5 CIDNP field dependences. In total, at least 15 protons and one nitrogen-14 in photoinduced biradical state of conjugate His-Gln(BP)-Tyr-Gly have non-zero HFCCs. This spin system is too large to be simulated within reasonable time using even an advanced personal computer. To reduce the computation time, some hyperfine interaction terms in Hamiltonian were approximated by an effective Schulten-Wolynes local field experienced only by the coupled electron^88,89^. Using this approximation, CIDNP field dependences of all protons were calculated separately, while including HFCCs of the majority of other magnetic nuclei in the amplitude of the Schulten-Wolynes local field. For example, CIDNP of six Tyr protons H2,6, H3,5, Hβ_1_ and Hβ_2_ was calculated using exact form of HFI, while the HFI with the magnetic nuclei of BP was approximated by Schulten-Wolynes local field $\:{B}_{SW}\:=\sqrt{\frac{2}{3}\cdot\:\sum\:{A}_{i}^{2}{S}_{i}\left({S}_{i}+1\right)}=\:$0.54 mT, where $\:{A}_{i}$ and $\:{S}_{i}$ are the HFCC and spin of the *i*-th nucleus of BP.

### DFT calculations

Quantum chemical calculations were performed with the ORCA program package (version 5.0.2)^90,91^. The TPSSh/def2-TZVPP^81,82^ method was used to optimize the geometries of anion and ketyl radicals of 4-amino benzophenone. The 7 water molecules forming hydrogen bonds with C = O and -NH_2_ groups (3 near the C = O group and 4 near the NH_2_ group) are taken into account explicitly with the def2-TZVP basis^82^. To calculate their HFCCs and g-factors of the anion and the ketyl radical of 4-aminobenzophenone, the TPSSh/ma-def2-QZVPP method was used^81,92^ with def2-TZVPP basis for water molecules. The HFCCs and g-factor of 4-amino benzophenone radical were calculated to compare them with the literature; also these data were compared with HFCCs calculated for His-Gln(BP)-Tyr-Gly biradical to ensure the correctness of the method choice.

The geometry of the biradical in its triplet state formed from His-Gln(BP)-Tyr-Gly conjugate was optimized by the TPSS/def2-SVP method, 11 water molecules were taken into account (primarily near the oxygen atoms of Tyr, BP, and CO_2_-) explicitly. The TPSSh functional was used to calculate the HFCCs, the ma-def2-QZVPP basis was used for the tyrosine -CH_2_-C_6_H_4_-O and benzophenone -CO-NH-C_6_H_4_-CO-C_6_H_5_ fragments, the def2-SVP basis was used for the rest of the peptide and water molecules. SMD solvation model^83^ with water as a solvent was used in all cases. Results of DFT calculation are shown in Supporting Information.

## Electronic supplementary material

Below is the link to the electronic supplementary material.

Supplementary Material 1

## Data Availability

The authors declare that the data supporting the findings of this study are available within the paper and its Supplementary Information files. Should any raw data files be needed in another format they are available from the corresponding author upon reasonable request. Source data are provided with this paper.
