# Supplementary material for: CIDNP study of photoinduced electron transfer in His-Glu-Tyr-Gly peptide and its conjugate His-Gln(BP)-Tyr-Gly
Source: Sci Rep. 2025 Jun 20;15:20135. doi: 10.1038/s41598-025-04831-6 (PMC12181371; doi:10.1038/s41598-025-04831-6)
Supplement: Supplementary file 1 — Supplementary Material 1 [file 41598_2025_4831_MOESM1_ESM.docx]

Supporting Information to

CIDNP study of photoinduced electron transfer in His-Glu-Tyr-Gly peptide and its conjugate His-Gln(BP)-Tyr-Gly

*Natalya N. Fishman^1§^, Kevin Herr^2§^, Olga B. Morozova^1^, Ivan V. Zhukov^1^, Maksim P. Geniman^1,3^, Martin Brodrecht^2^, Till Wissel^2^, Gerd Buntkowsky^2*^ and Alexandra V. Yurkovskaya^1*^*

^1^International Tomography Center, Institutskaya 3a, 630090 Novosibirsk, Russia

^2^Institute of Physical Chemistry, Technical University Darmstadt, Peter-Grünberg-Str. 8, 64287, Darmstadt, Germany.

^3^Novosibirsk State University, 2 ul. Pirogova, 630090 Novosibirsk, Russia

Email: [gerd.buntkowsky@chemie.tu-darmstadt.de](mailto:gerd.buntkowsky@chemie.tu-darmstadt.de)

[yurk@tomo.nsc.ru](mailto:yurk@tomo.nsc.ru)

§: both authors contributed equally





**Figure** **S1.** Protonation states of the His-Gln(BP)-Tyr-Gly conjugate together with the acidity constants.





**Figure** **S2.** pH^*^-dependence of chemical shift of H3,5 of Tyr residue in the peptide His-Gln(BP)-Tyr-Gly. Chemical shift was taken from CIDNP spectra obtained at 100 μs after the laser pulse irradiation of the peptide solution in D_2_O. Under these conditions, CIDNP intensity corresponds to a steady-state value, the signal of H2 of His decays to zero and does not overlap with the signal of H3,5 of Tyr. Determined p*K*_a_^*^=10.8. Corresponding p*K*_a_=10.4.





**Figure** **S3.** CIDNP kinetics for (a) proton H2 of the His residue (circles), (b) H3,5 protons of the Tyr residue (triangles), and (c) H6,6′ protons of TCBP (squares), obtained during the photoreaction between 2 mM TCBP and 20 mM (open symbols) or 40 mM (solid symbols) of the peptide His-Glu-Tyr-Gly at pH 8.8. Absolute value of CIDNP (emission) is plotted for the protons of Tyr. Dashed line – calculated CIDNP kinetics without taking into account electron transfer from tyrosine residue to histidine radical.





**Figure** **S4**. (top) 400 MHz ^1^H NMR spectrum (32 scans), obtained for the 0.12 mM solution of His-Gln(BP)-Tyr-Gly in D_2_O. (middle and bottom) 400 MHz ^1^H CIDNP spectra (16 scans), taken after the irradiation of the 0.12 mM solution of His-Gln(BP)-Tyr-Gly in D_2_O at *B*_pol_ = 7 mT (middle) or *B*_pol_ = 1 mT (bottom). The optical density was 1.4 cm^-1^ at 265 nm. The irradiation time was 3 s. The temperature was 25°C. The pH value of aqueous solution was 11.8.








**Figure** **S5**. ^1^H CIDNP field dependencies for Bp, Tyr and His protons of His-Gln(BP)-Tyr-Gly obtained in photoreactions of 0.12 mM of His-Gln(BP)-Tyr-Gly in D_2_O. The optical density was 1.4 cm^-1^ at 265 nm. The irradiation time was 3 s per scan. The pH values of aqueous solution were 8.7 and 11.8.

**Table S1.** Parameters used to model the CIDNP field dependence of His-Gln(BP)-Tyr-Gly in D_2_O, pH 3.9.

| **Symbol** | **Description** | **Value** |
| --- | --- | --- |
| ***Optimization parameters*** | | |
| $2J_{ex}$ | Amplitude of exchange interaction. The corresponding term in Hamiltonian is defined as: $\hat{H}_{ex}=-J_{ex}\left( \frac{1}{2}+2{\hat{\vec{S}}}_{1}\cdot{\hat{\vec{S}}}_{2} \right)$ | -8.78 mT |
| $R_{Jex}$ | Rate constant characterizing the magnitude of fluctuations of the exchange interaction. These fluctuations cause the electron spin singlet-triplet relaxation transitions with rate $W= 1/2\cdot{R_{Jex}}^{2}\cdot\tau_{Jex}\cdot J(\omega)$, where $J\left( \omega\right)= 1/\left( 1-\omega{\cdot\tau}_{Jex} \right)$ is the spectral density function of the fluctuations. | 1330 ns^-1^ |
| $T_{1e}$ | Relaxation time of individual electron spins defined for simple Bloch model in the extreme narrowing regime, where $T_{1e} = T_{2e}$. | 43.4 ns |
| ***Other parameters*** | | |
| $\tau_{Jex}$ | Correlation time of the fluctuations of the exchange interaction. | 1 ps |
| $P_{s}$ | Fraction of singlet-born biradicals. | 0 |
| $k_{S}$ | Recombination rate from singlet state of biradical | 1 ns^-1^ |
| $k_{T}$ | Recombination rate from triplet state of biradical | 0.001 ns^-1^ |
| $g_{1}$ | g-factor of TyrO^•^ radical | 2.0041 [b] |
| $g_{2}$ | g-factor of BP^•–^  radical [a] | 2.0035 |
| $a_{H2,6}$ | H2,6 HFCC in TyrO^•^ radical | 0.15 mT [b] |
| $a_{H3,5}$ | H3,5 HFCC in TyrO^•^ radical | -0.615 mT [b] |
| $a_{H\beta_{1},\beta_{2}}$ | H_β1, β2_  HFCC in TyrO^•^ radical | 0.77 mT [b] |
| $a_{N}$ | nitrogen-14 HFCC in 4-amino- BP^•–^  radical [a] | -0.066 mT |
| $a_{H-ortho}$ | H-*ortho* HFCC in 4-amino- BP^•–^  radical [a] | -0.3 mT |
| $a_{H-meta}$ | H-*meta* HFCC in 4-amino- BP^•–^  radical [a] | 0.122 mT |
| $a_{H-para}$ | H-*para* HFCC in 4-amino- BP^•–^  radical [a] | -0.38 mT |

[a] calculated using DFT method

[b] Tomkiewicz, M.; McAlpine, R. D.; Cocivera, M., Photooxidation and decarboxylation of tyrosine studied by EPR and CIDNP [chemically-induced dynamic nuclear polarization] techniques. *Can. J. Chem.* **1972**, 50, 3849-56.

**Calculated HFCCs and anisotropy parameters in the biradical of His-Gln(BP)-Tyr-Gly conjugate**





**Figure** **S6**. 4-aminobenzophenone radicals (anion, 4-I, and ketyl, 4- II) with proton numeration.

**Table S2.** The isotropic part of HFCCs and g-factor value, calculated for 4-aminobenzophenone radicals (anion, 4-I, and ketyl, 4- II)

|  | g | H-2 | H-3 | N-4 | H-NH­_2_ | H-5 | H-6 | H-2’ | H-3’ | H-4’ | H-5’ | H-6’ |
| --- | --- | --- | --- | --- | --- | --- | --- | --- | --- | --- | --- | --- |
| 4-I | 2.00346 | -0.306 | 0.107 | 0.129 | 0.092 | 0.118 | -0.270 | -0.354 | 0.139 | -0.436 | 0.121 | -0.322 |
| 4-II | 2.00303 | -0.313 | 0.116 | 0.163 | 0.048 | 0.118 | -0.295 | -0.394 | 0.159 | -0.451 | 0.155 | -0.382 |

**Table S3.** The isotropic and anisotropic parts of HFCCs, calculated for biradical of His-Gln(BP)-Tyr-Gly conjugate in its triplet state. The anisotropy tensor has three eigenvalues: a_x_, a_y_, a_z_. The anisotropy parameter p_aniso_ (in mT): $p_{\mathrm{aniso}}=\sqrt{\left( a_{\mathrm{iso}}-a_{x} \right)^{2}+\left( a_{\mathrm{iso}}-a_{y} \right)^{2}+\left( a_{\mathrm{iso}}-a_{z} \right)^{2}}$; $a_{\mathrm{iso}}=\left( a_{x}+a_{y}+a_{z} \right)/3$.

|  |  | a_iso_, mT | p_aniso_, mT |
| --- | --- | --- | --- |
| tyrosine | 3,5 | -0.618 | 0.494 |
|  | 2,6 | 0.176 | 0.170 |
|  | β-1 | 0.940 | 0.168 |
|  | β-2 | 0.292 | 0.170 |
| 4-aminobenzophenone | 2 | -0.316 | 0.156 |
|  | 3 | 0.130 | 0.098 |
|  | 5 | 0.120 | 0.084 |
|  | 6 | -0.288 | 0.160 |
|  | N | -0.066 | 0.012 |
|  | H (NH_2_) | 0.212 | 0.062 |
|  | 2’ | -0.324 | 0.158 |
|  | 3’ | 0.124 | 0.094 |
|  | 4’ | -0.380 | 0.314 |
|  | 5’ | 0.112 | 0.088 |
|  | 6’ | -0.288 | 0.170 |

**Synthesis of the His-Glu-Tyr-Gly peptide and His-Gln(BP)-Tyr-Gly conjugate**

**Synthesis of Fmoc-*NH*-Glu(*NH*-BP)-O*^t^*Bu 3**

**3**

The synthesis is based on experiments from Ikeda et al.^[1]^

2.39 g (1.1 equiv., 5.5 mMol) Fmoc-*NH*-Glu(OH)OtBu is dissolved in 50 mL dichloromethane (DCM) and 1.01 g (1.1 equiv., 5.5 mMol) 1-Ethyl-3-(3-dimethylaminopropyl)carbodiimide hydrochloride (EDC⋅HCl) is added. Subsequently, 68 mg (0.11 equiv., 0.55 mMol) 4-Dimethylaminopyridine (DMAP) and 1.01 g (1 equiv., 5 mMol) 3-Aminobenzophenone are subjoined. The solution was stirred for 12 h at ambient temperature and washed with 0.5 M hydrochloric acid, saturated sodium hydrogencarbonate and saturated sodium chloride solution and dried with sodium sulfate. The solvent was removed at 40 °C with reduced pressure and resulted in 3.4 g of crude product as a yellow powder.

**HPLC**: t_R_ = 7,4 min; **ESI-MS**: [M+H]^+^ = 605,3 m/z (calculated 605,3 m/z).


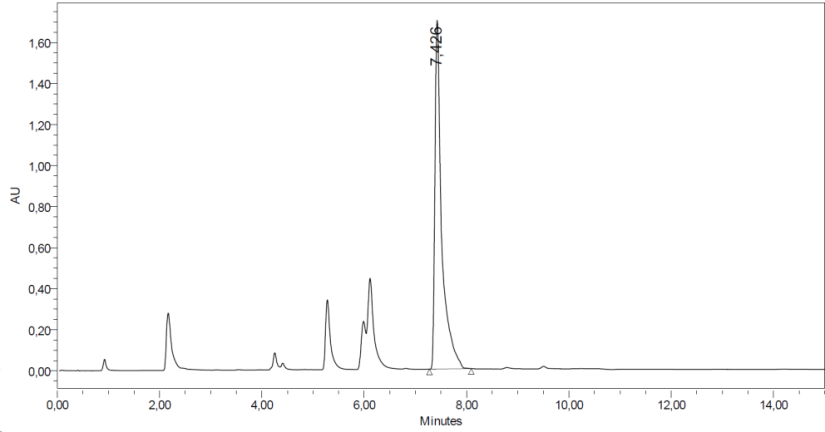


**Figure S7**. HPLC analysis of Fmoc-NH-Glu(NH-BP)-O^t^Bu **3** at 214 nm. The gradient was raised from 20 to 80 % of eluent B in 10 min with a flowrate of 2 mL/min.


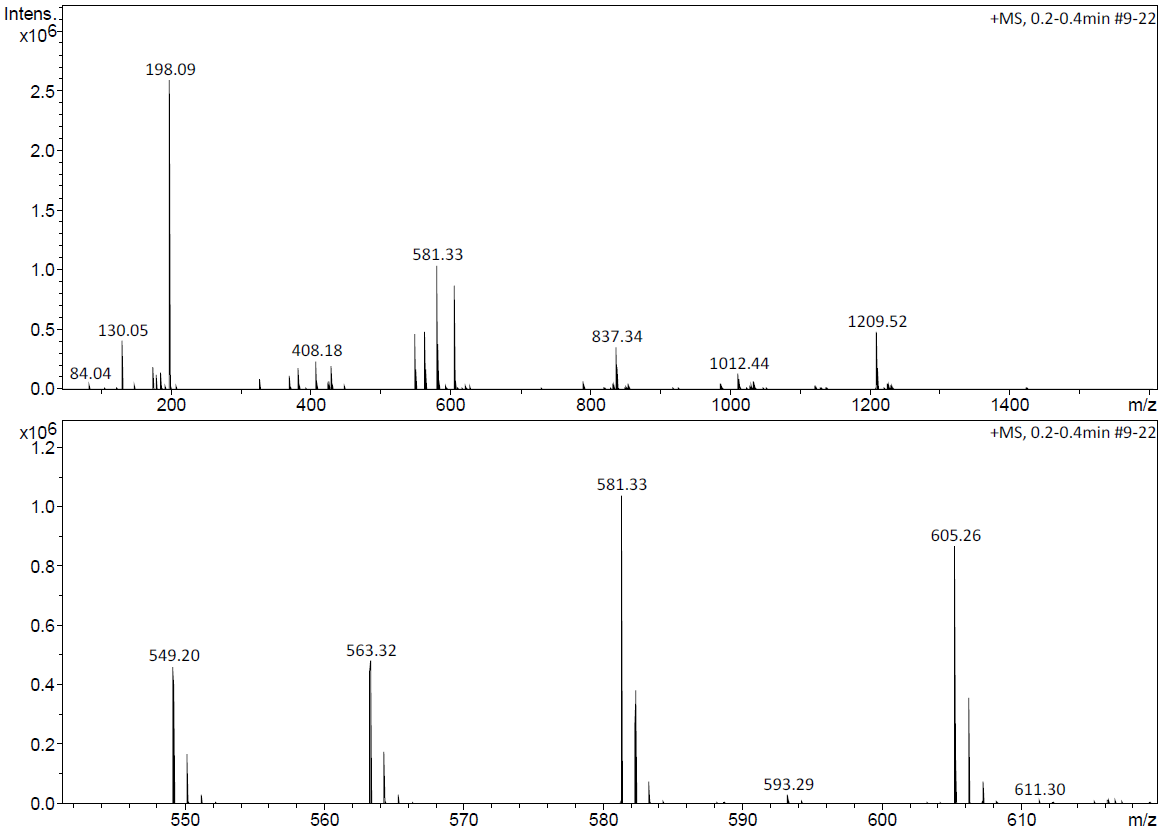


**Figure S8**.ESI-MS of Fmoc-NH-Glu(NH-BP)-O^t^Bu **3**.

**Synthesis of Fmoc-*NH*-Glu(*NH*-BP)-OH 4**

**4**

The synthesis is based on the protocol of Koivisto et al.^[3]^ 3.4 g (1 equiv., 5.0 mMol) Fmoc-*NH*-Glu(*NH*-BP)-O*^t^*Bu were dissolved in 35 mL DCM, cooled to 0 °C and 35.9 mL (89 equiv., 445 mMol) cooled TFA (95 %) was slowly added. The color of the solution turned from yellow to green during the process. The solution was stirred for 1.5 h at ambient temperature and the solvent was removed under reduced pressure. The residue of solved in DCM, washed three times with deionized water and saturated sodium chloride solution (DCM:washing solution 3:1 (v/v)) each and dried with sodium sulfate. The solvent was removed at 40 °C with reduced pressure and the crude product was purified by column chromatography using a mix of hexane/ethyl acetate (2:1 v/v). 1.35 g Fmoc-*NH*-Glu(*NH*-BP)-OH was obtained as a yellow powder.

**HPLC**: t_R_ = 5,5 min; **ESI-MS**: [M+H]^+^ = 549,2 m/z (calculated 549,2 m/z), [2M+H]^+^ = 1097,4 m/z (calculated 1097,4 m/z); **^1^H-NMR** (DMSO-d6, 500 MHz): δ[ppm] = 12,70, 10,32, 7,85, 7,75, 7,72, 7,71, 7,68, 7,62, 7,52, 7,39, 7,31, 4,27, 4,20, 4,05, 2,50, 2,12, 1,91.


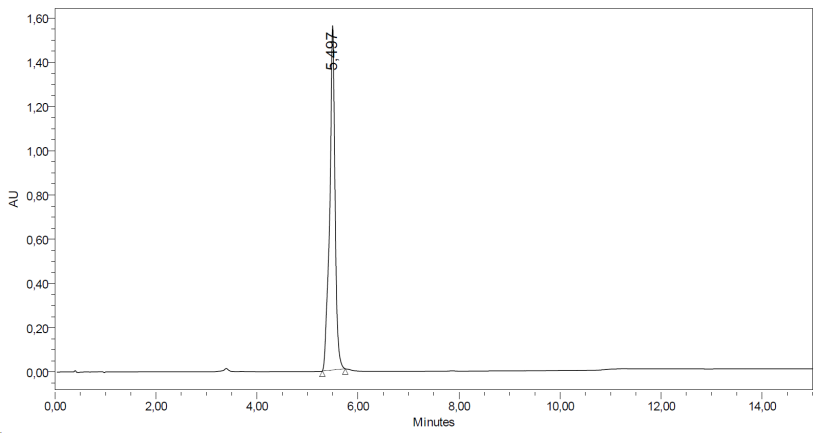


**Figure S9**. HPLC analysis of Fmoc-NH-Glu(NH-BP)-OH **4** at 214 nm. The gradient was raised from 20 to 80 % of eluent B in 10 min with a flowrate of 2 mL/min.


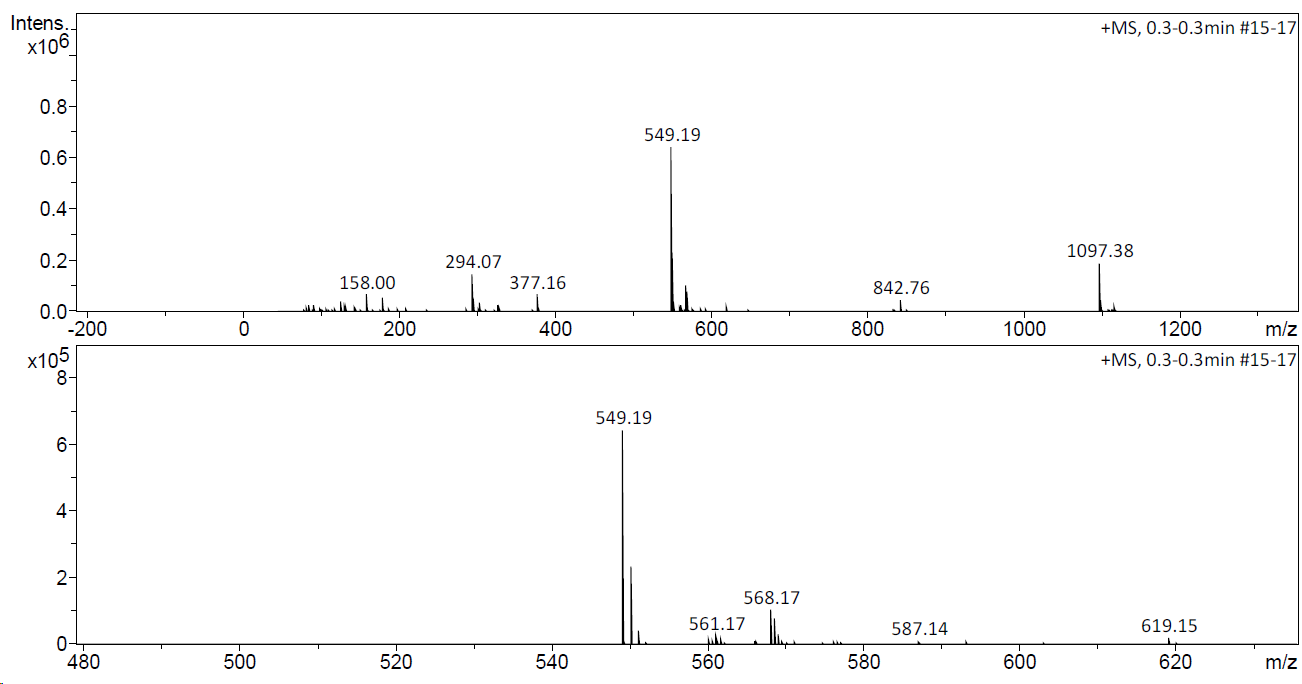


**Figure S10**. ESI-MS of Fmoc-NH-Glu(NH-BP)-OH **4**.


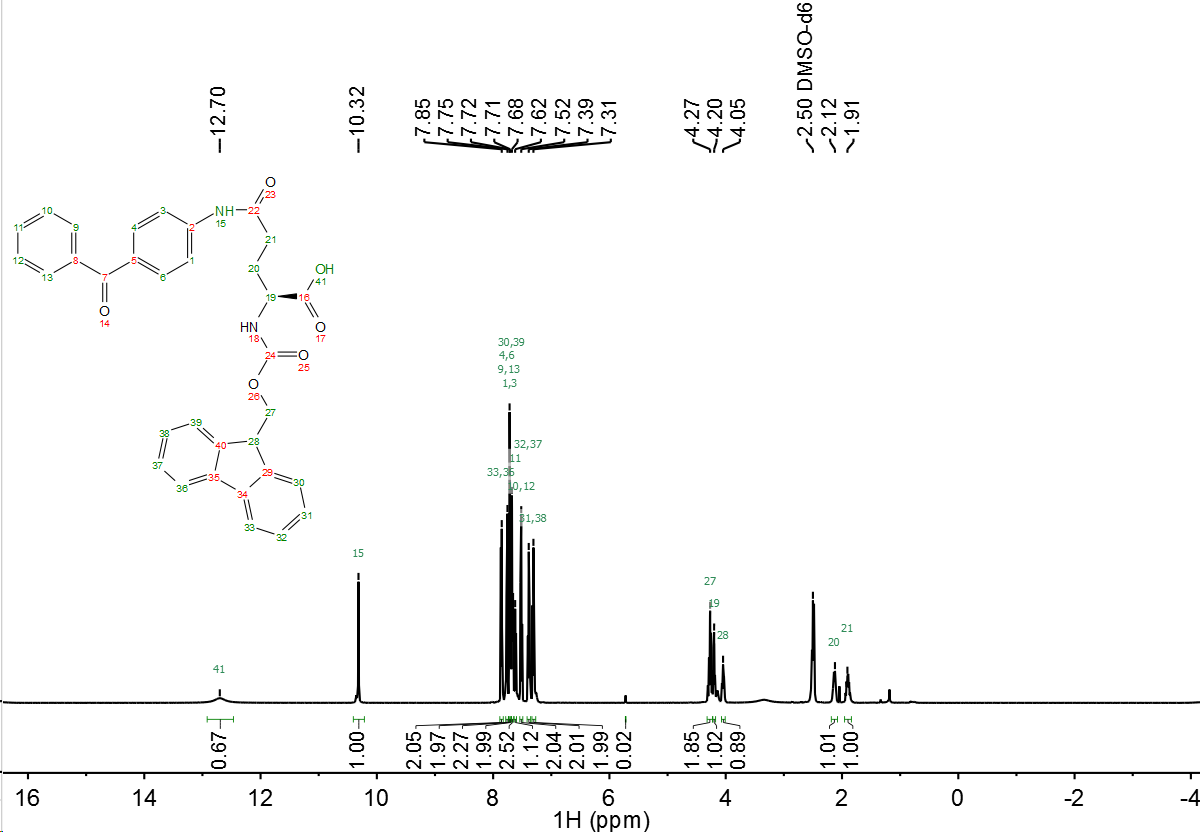


**Figure S11**. ^1^H-NMR spectrum of Fmoc-NH-Glu(NH-BP)-OH **4.**

**General protocol for the manual solid-state peptide synthesis**

The protocol is based on the work of Amblard et. al.^[4]^

In the synthesis protocol the terms “DMF I” and “DMF II” are used. Both terms refer to the solvent dimethylformamide (DMF) which are stored in different vessels since they are used in different washing processes. “DMF I” is used in the deprotection step of the Fmoc protection group and “DMF II” for washing during the coupling steps of the amino acids. This is to prevent contamination of “DMF II” since small amounts of the deprotection solution may have a bad influence during the synthesis and quality of the product.

**Activation of the resin:** First in a 10 mL syringe with a solid filter plate the 2-chlorotrityl chloride resin will be swelled in DMF II for 60 min and subsequently activated for 60 min with a solution of *N,N*-diisopropylethylamine (DIPEA) in DMF (1:7 mL per gram resin).

**Coupling:** To achieve the maximum amount of product and chain growth all coupling steps are done twice with an excess of the coupling amino acid. In general, all Fmoc amino acids will be dissolved in DMF II with a ratio of 12 mL DMF II per gram Fmoc amino acid. Only in the first coupling the Fmoc amino acid will be attached to the activated resin by adding DIPEA (2 equiv. in relation to 1 equiv. amino acid). Afterwards, additionally HATU (0.985 equiv. in relation to 1 equiv. amino acid) must be subjoined. The reaction solution is placed into the syringe with the resin and shaken for 30 min. Subsequently, the solution is removed from the syringe and the resin is washed 4 times with DMF II one minute each time. The coupling will be repeated with the same conditions one more time. When the coupling is performed twice the Fmoc protection group of the attached amino acid will be removed.

**Fmoc Deprotection:** The Fmoc protection group will be removed by shaking the resin 5 min and afterwards for 15 min with a 20 % piperidine in DMF solution. The resin is washed 3 times with DMF I and 3 times with DMF II one minute each time. After the deprotection the next coupling can be performed.

**Last coupling:** As a final washing procedure, the resin is washed 6 times with DCM and dried over high vacuum overnight.

**Cleavage of the resin:** The resin is mixed with a cleavage cocktail of trifluoroacetic acid (TFA)/anisole/triisopropylsilane (95/2.5/2.5 % v/v/v; 100 mg resin – 1 mL solution) and shaken for 3 h. The crude peptide is precipitated in 10 Veq (respectively to the cleavage cocktail) of 0°C cooled methyl *tert*-butyl ether (MTBE), incubated 30 min at -20 °C and washed each time once with cold MTBE and cold diethyl ether. The purification of the product was done by preparative HPLC and the removal of the solvent was achieved by lyophlilization.

**Synthesis of His-Glu-Tyr-Gly 1**

**1**

The synthesis of **1** was done as described and the weighing of all chemicals can be found in Table S4. The order of the coupling of the amino acids to 0.2 g of the resin (1 equiv., 1.46 mMolg^-1^) is the same as written in Table S4. 100 mg of peptide **1** was synthesized with a purity of > 99 %. In this case no purification by preparative HPLC was necessary.

**HPLC:** t_R_ = 10.1 min; **ESI-MS:** [M+H]^+^ = 505.2 m/z (calculated 505.2 m/z).

**Table S4.** Weighing of every coupling step for the synthesis of His-Glu-Tyr-Gly **1**.

| Coupling | Amino acid | Equivalent | Amount of Substance  [mmol] | Mass  [g] |
| --- | --- | --- | --- | --- |
| 1 | Fmoc-Gly-OH | 2 | 0.584 | 0.174 |
| 2 | Fmoc-Tyr(tBu)-OH | 2 | 0.584 | 0.268 |
| 3 | Fmoc-*NH*-L-Glu-OH ∙ H_2_O | 2 | 0.584 | 0.259 |
| 4 | Fmoc-His(Trt)-OH | 2 | 0.584 | 0.362 |


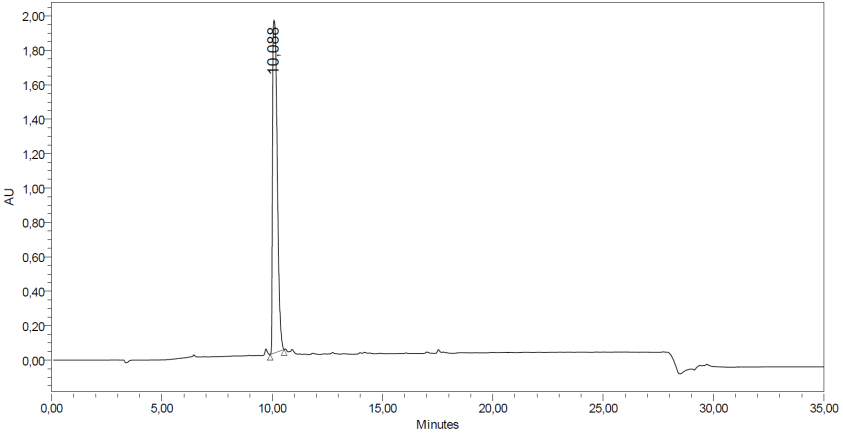


**Figure S12**. HPLC analysis of His-Glu-Tyr-Gly **1** at 214 nm. The gradient was raised from 0 to 50 % of eluent B in 25 min with a flowrate of 1 mL/min.


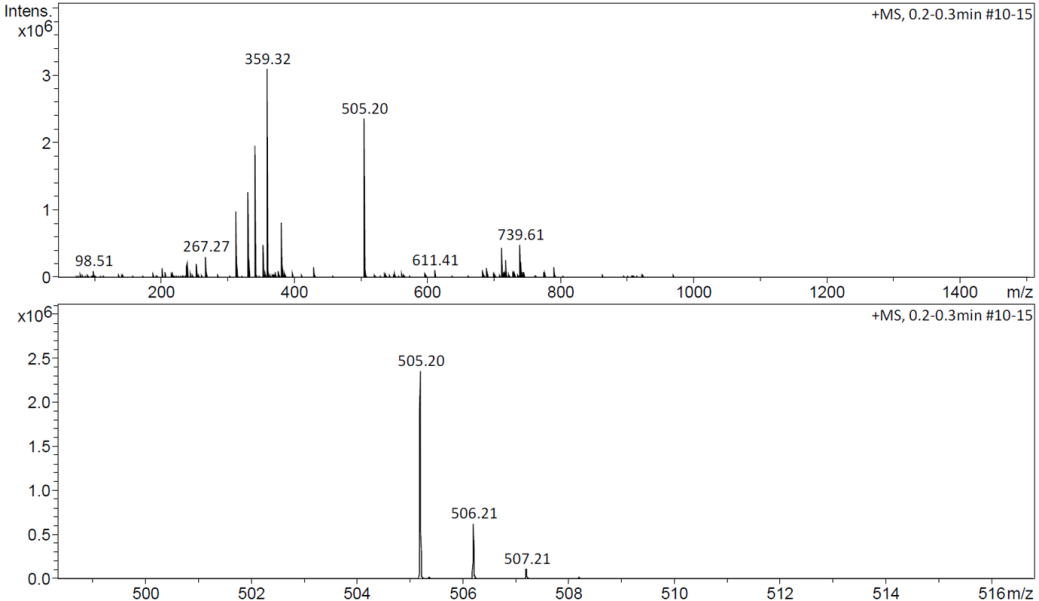


**Figure S13**. ESI-MS and zoom in of His-Glu-Tyr-Gly **1**.

**Synthesis of His-Gln(BP)-Tyr-Gly 2**

**2**

The synthesis of **2** was done as described and the weighing of all chemicals can be found in Table S5. The order of the coupling of the amino acids to 0.3 g of the resin (1 equiv., 1.46 mMolg^-1^) is the same as written in Table S5. 290 mg of the crude peptide was synthesized and purified via HPLC (flowrate 9 mL/min; gradient 0 to 50 % of eluent A in 60 min) resulting in 101 mg of the purified peptide **2** with a purity of > 99 %.

**HPLC:** t_R_ = 20.6 min; **ESI-MS:** [M+H]^+^ = 684.3 m/z (calculated 684.3 m/z).

**Table S5.** Weighing of every coupling step for the synthesis of His-Gln(BP)-Tyr-Gly **2**.

| Coupling | Amino acid | Equivalents | Amount of Substance  [mmol] | Mass  [g] |
| --- | --- | --- | --- | --- |
| 1 | Fmoc-Gly-OH | 2 | 0.876 | 0.26 |
| 2 | Fmoc-Tyr(tBu)-OH | 2 | 0.876 | 0.403 |
| 3 | Fmoc-*NH*-L-Glu(*NH*-BP)-OH **4** | 2 | 0.876 | 0.481 |
| 4 | Fmoc-His(Trt)-OH | 2 | 0.876 | 0.543 |


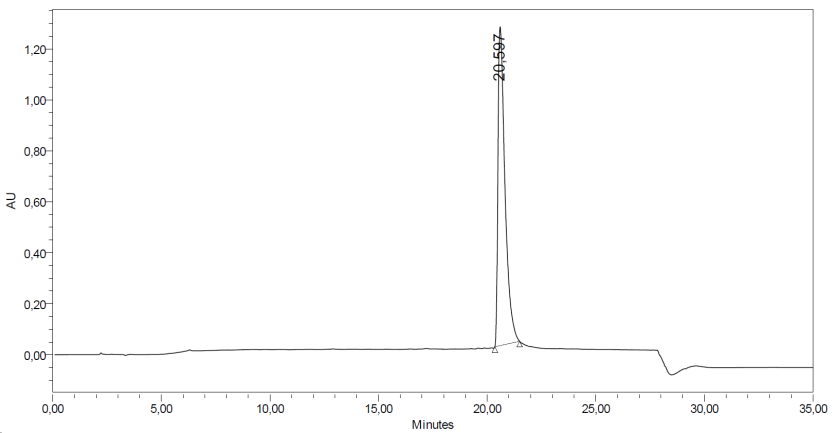


**Figure S14**. HPLC analysis of His-Gln(BP)-Tyr-Gly **2** at 214 nm. The gradient was raised from 0 to 50 % of eluent B in 25 min with a flowrate of 1 mL/min.


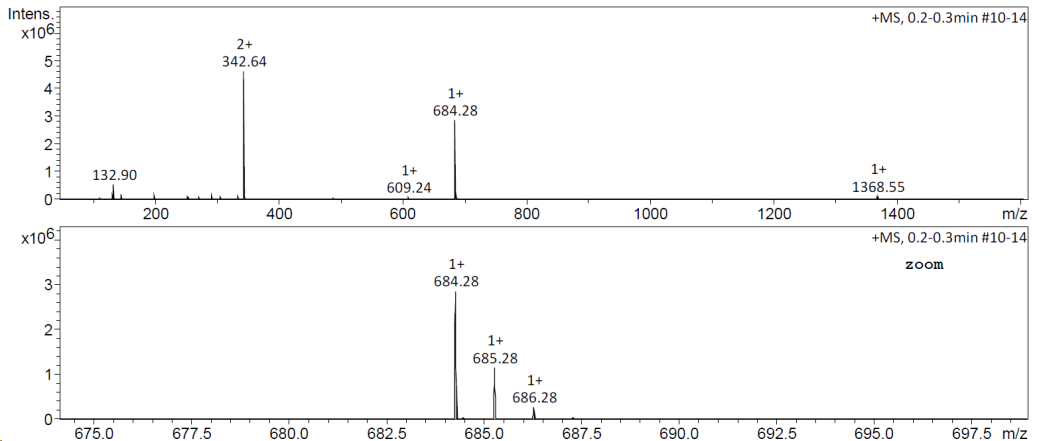


**Figure S15**. ESI-MS and zoom in of His-Gln(BP)-Tyr-Gly **2**.

**Mass spectrometry (MS)**

Spectra were recorded by the analytical department of the TU Darmstadt with an Esquire-LC device from *Bruker* using the ESI method.

**High performance liquid chromatography (HPLC)**

HPLC analysis was used for analytical and preparative separation with a detection wavelength of 214, 254 and 301 nm and eluents A (0.1 % aq. TFA) and B (99.9 % acetonitrile with 0.1 % TFA). The specifications for the separation are described in the experimental part for each product.

Specifically, for the analytical separation a device from *Waters* (*Waters Alliance* e2695/*Waters* 2998 PDA detector) and a *Nucleosil* 100-5 C18 column (particle size: 5 μm; pore size 100 Å) were applied.

The preparative separation was done with a device from *Waters* (*Waters* 600/*Waters* 717 plus autosampler/*Waters* 996 PDA detector and *Waters Alliance* e2695/*Waters* 2998 PDA detector) with a *Knauer Multikrom* RP18 20300 mm column (particle size: 5mm; pore size 100 Å).

**NMR**

The liquid NMR analysis was performed at room temperature with a DRX 500 (^1^H-NMR at 500 MHz) device from *Bruker*. The chemical shift is displayed in ppm, the solvent signal serves as an internal standard and the analysis was done by *MestReNova* software from *Mestrelab* Research.

**Literature to the synthesis:**

[1] A. Ikeda, K. Terada, M. Shiotsuki, F. Sanda, *J. Polym. Sci. A Polym. Chem.* **2011**, *49*, 3783–3796.

[2] M. Brodrecht, K. Herr, S. Bothe, M. de Oliveira, T. Gutmann, G. Buntkowsky, *ChemPhysChem* **2019**, *20*, 1475–1487.

[3] J. J. Koivisto, E. T. T. Kumpulainen, A. M. P. Koskinen, *Organic & biomolecular chemistry* **2010**, *8*, 2103–2116.

[4] M. Amblard, J.-A. Fehrentz, J. Martinez, G. Subra, *Mol. Biotechnol.* **2006**, *33*, 239–254.
